# The CNOT4 Subunit of the CCR4‐NOT Complex is Involved in mRNA Degradation, Efficient DNA Damage Repair, and XY Chromosome Crossover during Male Germ Cell Meiosis

**DOI:** 10.1002/advs.202003636

**Published:** 2021-03-16

**Authors:** Xing‐Xing Dai, Yu Jiang, Jia‐Hui Gu, Zhi‐Yan Jiang, Yun‐Wen Wu, Chao Yu, Hao Yin, Jue Zhang, Qing‐Hua Shi, Li Shen, Qian‐Qian Sha, Heng‐Yu Fan

**Affiliations:** ^1^ MOE Key Laboratory for Biosystems Homeostasis & Protection and Innovation Center for Cell Signaling Network Life Sciences Institute Zhejiang University Hangzhou 310058 China; ^2^ College of Life Science Zhejiang University Hangzhou 310058 China; ^3^ First Affiliated Hospital of USTC Hefei National Laboratory for Physical Sciences at Microscale School of Basic Medical Sciences Division of Life Sciences and Medicine CAS Center for Excellence in Molecular Cell Science University of Science and Technology of China Hefei 230027 China; ^4^ Clinical Research Center for Reproduction and Genetics in Hunan Province Reproductive and Genetic Hospital of CITIC‐XIANGYA Changsha 410008 China; ^5^ Fertility Preservation Laboratory Reproductive Medicine Center Guangdong Second Provincial General Hospital Guangzhou 510317 China

**Keywords:** germ cell development, homologous recombination, mRNA stability, polyadenylation, spermatogenesis

## Abstract

The CCR4‐NOT complex is a major mRNA deadenylase in eukaryotes, comprising the catalytic subunits CNOT6/6L and CNOT7/8, as well as CNOT4, a regulatory subunit with previously undetermined functions. These subunits have been hypothesized to play synergistic biochemical functions during development. *Cnot7* knockout male mice have been reported to be infertile. In this study, viable *Cnot6*/*6l* double knockout mice are constructed, and the males are fertile. These results indicate that CNOT7 has CNOT6/6L‐independent functions in vivo. It is also demonstrated that CNOT4 is required for post‐implantation embryo development and meiosis progression during spermatogenesis. Conditional knockout of *Cnot4* in male germ cells leads to defective DNA damage repair and homologous crossover between X and Y chromosomes. CNOT4 functions as a previously unrecognized mRNA adaptor of CCR4‐NOT by targeting mRNAs to CNOT7 for deadenylation of poly(A) tails, thereby mediating the degradation of a subset of transcripts from the zygotene to pachytene stage. The mRNA removal promoted by the CNOT4‐regulated CCR4‐NOT complex during the zygotene‐to‐pachytene transition is crucial for the appropriate expression of genes involved in the subsequent events of spermatogenesis, normal DNA double‐strand break repair during meiosis, efficient crossover between X and Y chromosomes, and ultimately, male fertility.

## Graphical abstract

In the CCR4‐NOT complex, CNOT4 facilitates CNOT7‐mediated deadenylation/degradation of mRNAs from the zygotene to pachytene stage of spermatogenesis through its RNA recognition motif. CNOT4‐dependent mRNA removal in meiotic prophase is required for the appropriate expression of genes involved in the subsequent events of spermatogenesis, DSB repair during meiosis, and efficient crossover formation between X and Y chromosomes.

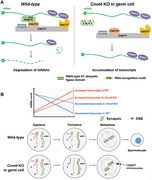

## 1 Introduction

Modification by the elongation and shortening of the poly(A) tail is the most common type of post‐transcriptional mRNA regulation in eukaryotic cells.^[^ ^1^ ^]^ The evolutionarily conserved CCR4‐NOT complex is a major RNA deadenylase in eukaryotes.^[^ ^2^ ^]^ The mammalian CCR4‐NOT deadenylase is a multi‐subunit complex that includes CNOT1, which is a scaffold protein that contains docking domains for several other subunits and two structurally distinct catalytic subunits: CNOT6 (or its homolog CNOT6L) and CNOT7 (or its homolog CNOT8).^[^ ^3^ ^]^ In vitro, CNOT6/6L and CNOT7/8 synergistically catalyze the deadenylation of synthetic poly(A)s.^[^ ^4^, ^5^, ^6^ ^]^ However, genetic evidence obtained from yeast and mice with genetic mutations suggests that these subunits are not required for mRNA deadenylation in vivo.^[^ ^7^, ^8^ ^]^ Instead, each has cell type‐ and developmental process‐specific physiological functions.

The human CCR4‐NOT complex contains seven stable core components and several relatively loosely associated regulatory subunits such as CNOT4, which has received particular attention as an E3 ubiquitin ligase,^[^ ^9^, ^10^ ^]^ CNOT4 contains a RING domain near its N‐terminus that mediates the interaction between CNOT4 and several ubiquitin‐conjugating enzymes (E2).^[^ ^11^ ^]^ Diverse proteins in yeast and human cell lines have been reported to be ubiquitination targets of CNOT4, including the master regulator of meiosis MEI2,^[^ ^12^ ^]^ co‐translational quality control factor ABCE1,^[^ ^13^ ^]^ histone H3 lysine‐9 demethylase JHD2,^[^ ^14^ ^]^ transcriptional coactivator YAP1,^[^ ^15^ ^]^ and the ribosomal protein RPS7A.^[^ ^16^ ^]^ In addition to its potential role in mediating protein ubiquitination, CNOT4 also contains an RNA recognition motif (RRM), although its RNA‐binding ability has not been firmly demonstrated in vivo.^[^ ^17^ ^]^ This raises the question of whether CNOT4 functions as a substrate mRNA adaptor of CCR4‐NOT deadenylase in metazoans. Moreover, the mRNAs that CNOT4 can bind in vivo and the physiological function of CNOT4 in animals have not yet been reported.

Studies in gene knockout mice indicated that CCR4‐NOT‐mediated mRNA decay is particularly important in germ cell development and the maternal‐to‐zygotic transition process. Whole body deletion of *Cnot6l* impairs maternal mRNA clearance during oocyte maturation, thereby causing female sterility,^[^ ^18^, ^19^ ^]^ The knockout of maternal *Btg4*, which is a gene encoding an oocyte‐specific adaptor protein of CNOT7 that is essential in mediating mRNA deadenylation, blocks zygotic development at the 1–2 cell stage,^[^ ^20^, ^21^ ^]^ However, knockout of *Cnot7* itself interrupts only spermatogenesis and not oogenesis, thereby causing only male infertility.^[^ ^22^ ^]^ Although the infertile phenotype of *Cnot7* KO mice was reported nearly two decades ago, the pertinent primary defects were not indicated, and the changes in the transcriptome of *Cnot7* KO spermatocytes were not analyzed. Altogether, we hypothesized that the diverse phenotypes of these knockout mice suggest that the two catalytic subunits of CCR4‐NOT may not necessarily function synergistically in vivo but have independent roles in different cell types. Furthermore, although BTG4 was identified as a mediator of CCR4‐NOT‐dependent mRNA clearance in oocytes and zygotes, the mRNA adaptor of CCR4‐NOT deadenylase in male germ cells remains elusive.

Mammalian spermatogenesis typically consists of three distinct phases: Mitosis, meiosis, and spermiogenesis. Meiosis I in spermatocytes progresses through the leptotene, zygotene, pachytene, and diplotene stages and finally enters the metaphase; programmed formation and repair of DNA double‐strand breaks (DSBs), pairing (synapsis) of paternal and maternal chromosomes, homologous recombination, and other landmark events occur, and eventually, crossovers are formed on the paired chromosomes to ensure accurate separation during anaphase I,^[^ ^23^, ^24^ ^]^ XY chromosomes are synapsed only in a small homologous region (pseudo‐autosomal region, PAR).^[^ ^25^ ^]^ The distinct properties of chromatin organization at the PAR are crucial for male meiosis;^[^ ^26^ ^]^ an erroneous separation of X and Y chromosomes leads to chromosomal aneuploidy, infertility, and Klinefelter syndrome.^[^ ^27^ ^]^ There are many differences between autosomes and XY chromosomes in the characteristics and regulatory mechanisms in the DSB formation/repair and chromosome pairing.^[^ ^28^ ^]^ The biological processes and protein factors that affect XY chromosome crossover have not been fully elucidated.

Spermatogenesis is regulated at both the transcriptional and post‐transcriptional levels. In spermatocytes, transcription is relatively inert in the leptotene and zygotene stages of meiosis, and transcription increases at the pachytene stage. However, the zygotene‐to‐pachytene transition is also associated with a wave of programmed mRNA degradation. According to a recent transcriptomic analysis, transcription slows during spermatogenesis, particularly between the zygotene to pachytene phase and spermiogenesis.^[^ ^29^ ^]^ The physiological significance of the marked changes in the transcriptome during the zygotene to pachytene phase requires further investigation. Terminal uridine transferase (TUT) 4 and 7 are proteins that promote mRNA degradation in eukaryotic cells;^[^ ^30^ ^]^ they promote the oligo(U)‐modification of mRNAs that contain a short poly(A) tail, which is followed by the recognition of the mRNAs for degradation.^[^ ^31^ ^]^ Moreover, TUT4 and TUT7 were reported to promote the degradation of mRNA from the zygotene to pachytene phase during spermatogenesis.^[^ ^29^ ^]^ Spermatogenesis of *Tut4* and *Tut7* double‐knockout males was arrested at the pachytene stage of meiosis I. The regulation mechanism of the transcriptome decline from the zygotene to pachytene phase during spermatogenesis has not yet been elucidated.

In this study, we demonstrated that CNOT4 has indispensable functions during germ cell meiosis in mice. *Cnot4* is essential for the development of post‐implantation embryos and is required for spermatogenesis. The CCR4‐NOT^CNOT4^ complex has previously unrecognized functions in DSB repair and crossover between XY chromosomes during meiosis.

## 2 Results

### 2.1 CNOT6/6L Catalytic Subunits of CCR4‐NOT are Dispensable for Most Developmental Events Including Spermatogenesis

To investigate the importance of CNOT6 and CNOT6L catalytic subunits in the CCR4‐NOT complex in vivo, we knocked out both *Cnot6* (Figure S1A,B, Supporting Information) and *Cnot6l* (*Cnot6/6l^–/–^*) in mice. Surprisingly, *Cnot6/6l^–/–^* mice were viable and healthy, indicating that *Cnot6/6l* is not essential for survival. *Cnot6/6l^–/–^* male mice had normal reproductive function (Figure S1C, Supporting Information), whereas *Cnot6/6l^–/–^* female mice were infertile as CNOT6L is essential for maternal mRNA clearance during oocyte meiotic maturation.^[^ ^18^ ^]^ We used an antibody that targets both CNOT6 and CNOT6L for western blots; the results indicated that CNOT6/6L was completely absent in *Cnot6/6l^–/–^* testicular lysates (Figure S1D, Supporting Information). Hematoxylin and eosin (H&E) staining indicated that the testicular seminiferous tubules in adult *Cnot6/6l^–/–^* mice had normal histology and spermatogenic cells were present at all developmental stages (Figure S1E, Supporting Information). The epididymides of 3‐month‐old *Cnot6/6l^–/–^* male mice were filled with spermatozoa (Figure S1E, Supporting Information). The amount of sperm stored in the epididymis was comparable between adult (3 months old) wild‐type (WT) and *Cnot6/6l* double knockout males (Figure S1F, Supporting Information). Therefore, in contrast to CNOT7, CNOT6 and CNOT6L are not required for spermatogenesis, indicating that the function of CNOT7 in mouse testis is CNOT6/6L‐independent.

### 2.2 Embryos with Knocked Out *Cnot4* Die after Implantation

It is unclear whether other proteins in the CCR4‐NOT complex, such as CNOT4, function synergistically with CNOT7 in terms of spermatogenesis regulation. CNOT4 is a regulatory subunit of CCR4‐NOT deadenylase. Therefore, we investigated the role of CNOT4 in mouse development, particularly spermatogenesis.

To determine *Cnot4* function in vivo, we established *Cnot4*‐floxed mice and crossed them with the *Stra8‐Cre* transgenic mice. From their offspring, we obtained a *Cnot4* knockout mouse strain with exon 2 deletion (266 bp) and a reading frame shift (Figure S2A, Supporting Information). We repeatedly mated heterozygous *Cnot4^+/−^* male and female mice but did not produce *Cnot4^–/–^* offspring mice (Figure S2B, Supporting Information), suggesting that *Cnot4^–/–^* embryos died during prenatal development.

Next, we determined the stage at which *Cnot4* knockout was lethal to embryos. Heterozygous *Cnot4^+/−^* female and male mice were mated; the preimplantation embryos were harvested by flushing the uterus at 4.5 days post coitus (dpc) and genotyped. The percentages of *Cnot4^+/+^*, *Cnot4^+/−^*, and *Cnot4^−/−^* blastocysts matched the Mendelian genetic laws (Figure S2C, Supporting Information). *Cnot4^/−^* embryos could develop into blastocysts and had normal morphology (Figure S2D, Supporting Information). Then, we collected the uteri of female mice that contained embryos that developed and degenerated at 11.5 dpc (Figure S2E, Supporting Information). Genotyping indicated that all the degenerated embryos were homozygous for the *Cnot4* mutation (Figure S2E, Supporting Information). We also performed western blotting using total protein from isolated WT and KO embryos. The results confirmed that CNOT4 protein was absent in KO embryos (Figure S2F, Supporting Information). Therefore, embryos with *Cnot4* knockout died after implantation and before 11.5 dpc of development.

### 2.3 *Cnot4^fl/fl^;Stra8‐Cre* Male Mice Exhibit Impaired Spermatogenesis and are Infertile

**Figure 1:**
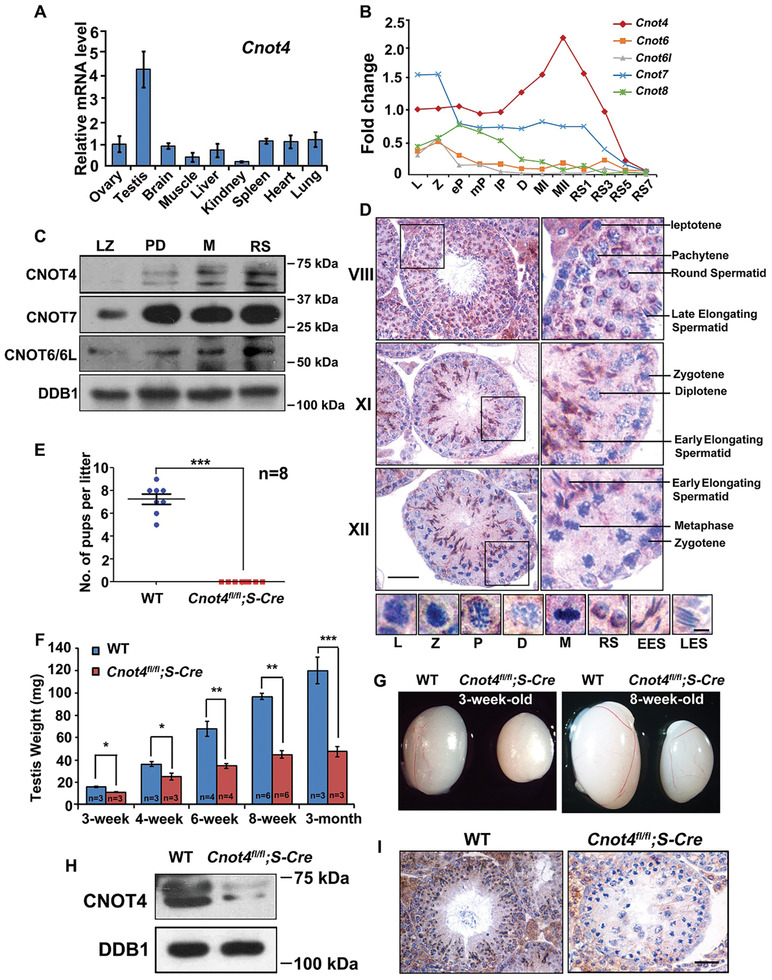
CNOT4 is expressed in mouse testis and is required for male fertility. A) Expression of *Cnot4* in different organs measured using quantitative reverse‐transcription PCR (RT‐qPCR) in tissue lysates. B) Relative mRNA expression of *Cnot4*, *Cnot7/8*, and *Cnot6/6l* in mouse spermatocytes extracted from published RNA sequencing results.^[^ ^32^ ^]^ L: leptotene; Z: zygotene; P: pachytene; D: diplotene; MI: meiosis I; MII: meiosis II; RS: round spermatid. C) Western blotting of CNOT4, CNOT7, and CNOT6/6L in spermatocytes isolated from testis of wild‐type (WT) mice by flow cytometry. Endogenous DDB1 was blotted as a loading control. LZ: leptotene and zygotene; PD: pachytene and diplotene; M: meiosis II; RS: round spermatid. D) Immunohistochemical staining of CNOT4 expression in spermatocytes. Long scale bar = 50 µm. Short scale bar = 5 µm. E) Fertility test results of WT and *Cnot4^fl/fl^;Stra8‐Cre* males. *N* = 8 males for each genotype. F) Testis weight of WT and *Cnot4^fl/fl^;Stra8‐Cre* mice. The numbers of male mice are indicated by *n*. G) Morphology of representative testes from the WT and *Cnot4^fl/fl^;Stra8‐Cre* mice. Scale bar = 1 mm. H) Western blotting of CNOT4 in the testis lysates of WT and *Cnot4^fl/fl^;Stra8‐Cre* mice. Endogenous DDB1 was blotted as a loading control. I) Immunohistochemical detection of knockout efficiency of CNOT4 in *Cnot4^fl/fl^;Stra8‐Cre* mice. Scale bar = 50 µm; error bars, SEM; *p < 0.05; **p < 0.01, ***p < 0.001; statistical significance values were determined with two‐tailed Student's *t*‐tests.

Quantitative RT‐PCR (RT‐qPCR) analyses using total RNA isolated from tissue lysates indicated that *Cnot4* had higher mRNA expression in the testis than in other organs (**Figure** **1**A). Spermatocyte transcriptome analysis revealed that *Cnot4* mRNA expression increases gradually in the meiotic prophase and decreases after metaphase I (MI) (Figure 1B).^[^ ^32^ ^]^ We isolated spermatocytes from 6‐week‐old male mice using flow cytometry and detected protein expression in spermatocytes at leptotene/zygotene, pachytene/diplotene, and metaphase stages and in haploid spermatocytes. According to the NCBI database (Gene ID: 53 621), CNOT4 has multiple isoforms, ranging from 572 to 710 aa. Western blot analysis demonstrated that at least two CNOT4 isoforms were simultaneously expressed in the testis (Figure 1C). CNOT4 expression gradually increased in spermatocytes during meiosis (Figure 1C; Figure S3A, Supporting Information). CNOT7 also accumulated, but protein expression of CNOT6/6L did not change significantly (Figure 1C; Figure S3A, Supporting Information). Immunohistochemical staining indicated that CNOT4 localized to the cytoplasm of spermatocytes; CNOT4 accumulated during meiosis and decreased rapidly in the late elongating spermatids (Figure 1D). We used the *Stra8‐cre* model to knock out *Cnot4* in male germ cells before they entered meiosis and investigated the role of CNOT4 in spermatogenesis (Figure S2A, Supporting Information).

*Cnot4^fl/fl^;Stra8‐Cre* males were sterile (Figure 1E). The testes of *Cnot4^fl/fl^;Stra8‐Cre* male mice were significantly smaller than those of the age‐matched WT male mice (Figure 1F,G). Western blot analysis indicated that CNOT4 levels were greatly reduced in the testes of *Cnot4^fl/fl^;Stra8‐Cre* male mice (Figure 1H and Figure S3B, Supporting Information). Furthermore, immunohistochemistry indicated that CNOT4 was absent in the germ cells of *Cnot4^fl/fl^;Stra8‐Cre* males (Figure 1I). These results also indicated that the CNOT4 antibody is specific for endogenous CNOT4.

**Figure 2:**
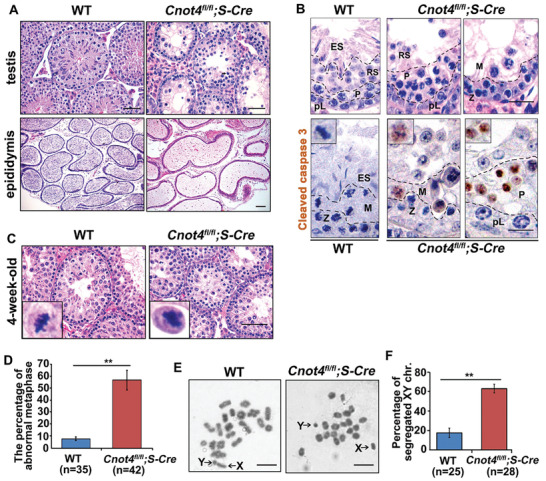
CNOT4 is required for spermatogenesis. A) Histology of the epididymis and testis from 6‐week‐old WT and *Cnot4^fl/fl^;Stra8‐Cre* mice by H&E staining. Scale bar = 50 µm. B) Sections of stage IX–X tubules from WT and *Cnot4^fl/fl^;Stra8‐Cre* mice stained with H&E and the immunohistochemical staining of cleaved caspase 3. Scale bar = 20 µm. C,D) H&E staining and percent metaphase I (MI)‐stage spermatocytes with lagged chromosomes in WT and *Cnot4^fl/fl^;Stra8‐Cre* mice. E) Giemsa staining of spermatocyte spreads of 17‐day‐old mice. Scale bar = 10 µm. F) Percent MI‐stage spermatocytes with segregated X and Y chromosomes. *n*, number of MI spermatocytes; error bars, SEM; **p < 0.01, determined by two‐tailed Student's *t*‐tests.

H&E staining indicated that the testicular lumina of 6‐week‐old wild‐type (WT) mice was filled with spermatocytes at various stages. In contrast, in the testicular lumen of age‐matched *Cnot4^fl/fl^;Stra8‐Cre* male mice, there were many large cavities and very few spermatocytes (**Figure** **2**A). In addition, normal sperm were not present in the epididymis of *Cnot4^fl/fl^;Stra8‐Cre* male mice (Figure 2A). Histological analysis and cleaved caspase 3 immunostaining of *Cnot4^fl/fl^;Stra8‐Cre* testes revealed spermatogenic arrest in metaphase I (Stages IX–X) accompanied by apoptosis (Figure 2B). We also applied H&E staining to the WT and *Cnot4*‐knockout 3‐week testes (Figure S3C, Supporting Information) and found that there were still abundant pachytene spermatocytes in the *Cnot4*‐knockout testes. Immunofluorescence staining for cleaved caspase 3, an apoptotic cell signal, indicated that the number of apoptotic spermatocytes was significantly increased in the *Cnot4*‐knockout testes at 4–6 weeks after birth (Figure S3D,E, Supporting Information).

In the WT male mice, cells in the first wave of spermatogenesis entered the pachytene stage, 17–18 days after birth, progressed to MI at 4 weeks.^[^ ^33^ ^]^ The testicular histology of 17‐day‐old *Cnot4^fl/fl^;Stra8‐Cre* males was similar to that of WT mice (Figure S3C, Supporting Information). Markers of meiotic prophase, including phosphorylated histone H2AX (pH2AX) and SYCP3, as well as, the late pachytene cell marker histone H1t, were detected in the testicular tubules (Figure S3F, Supporting Information). Normal MI spermatocytes were seen in the testes of 4‐week‐old WT male mice (Figure 2C). The spermatocytes of *Cnot4^fl/fl^;Stra8‐Cre* males developed until the MI stage, but their chromosomes failed to align at the spindle middle plate (Figure 2C,D). Giemsa staining of the germ cell chromosome spread showed that many *Cnot4^fl/fl^;Stra8‐Cre* spermatocytes at MI contained precociously separated XY chromosomes (Figure 2E). The percentage of segregated XY chromosomes in *Cnot4*‐null spermatocytes at MI was significantly higher than that in the WT cells (Figure 2F).

### 2.4 CNOT4 is Required for XY Chromosome Pairing

**Figure 3:**
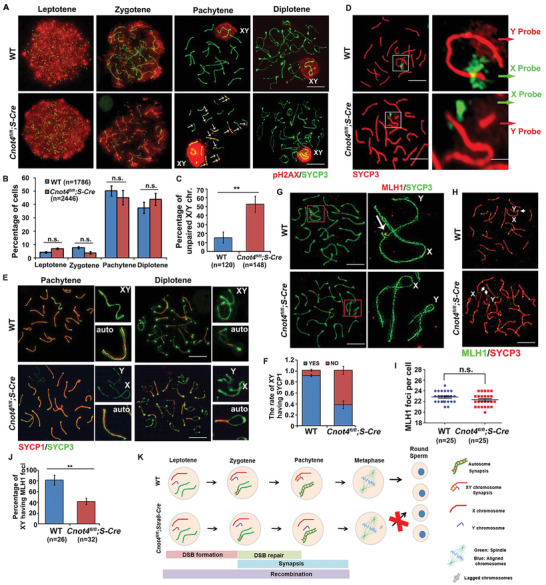
Abnormal XY chromosome pairing in the spermatocytes of *Cnot4^fl/fl^;Stra8‐Cre* male mice. A) Immunofluorescence staining of SYCP3 and pH2AX of spermatocyte spreads from WT and *Cnot4^fl/fl^;Stra8‐Cre* mice. Scale bar = 10 µm. B) Percent of spermatocytes at the leptotene, zygotene, pachytene, and diplotene stages in the WT and *Cnot4^fl/fl^;Stra8‐Cre* mice. C) Percent of pachytene‐stage spermatocytes with separated X and Y chromosomes. D) DNA FISH labeling of X and Y chromosomes using indicated fluorescent probes. Red: Y probe and SYCP3, Green: X probe. X and Y chromosomes are framed. E) Immunofluorescence staining of SYCP3 and SYCP1 of spermatocyte spreads prepared from WT and *Cnot4^fl/fl^;Stra8‐Cre* mice. Scale bar = 10 µm; auto, autochromosome. F) Rates of pachytene spermatocytes negative and positive for SYCP1 signals on X and Y chromosomes. Immunofluorescence staining of SYCP3 and MLH1 in spermatocyte spreads from WT and *Cnot4^fl/fl^;Stra8‐Cre* mice. Images taken by G) ultra‐high‐resolution microscopy or H) normal confocal microscopy. In (G), the arrow indicates the MLH1 focus, and XY chromosomes are framed. In (H), XY chromosomes are indicated by arrows. Scale bar = 10 µm. I) Average numbers of MLH1 foci in each pachytene spermatocyte. J) Percentage of spermatocytes with MLH1 foci on XY chromosomes of WT and *Cnot4^fl/fl^;Stra8‐Cre* mice. K) Graphic illustration showing spermatogenic defects of *Cnot4^fl/fl^;Stra8‐Cre* mice. *n*, number of cells; error bars, SEM; n.s., non‐significant; **p < 0.01 determined by two‐tailed Student's *t*‐tests.

The abnormal characteristics of *Cnot4‐*deleted spermatocytes in MI could be due to defects in the meiosis I prophase. Thus, we evaluated the abnormality of *Cnot4*‐null spermatocytes in meiotic prophase. We examined the testes of WT and *Cnot4^fl/fl^;Stra8‐Cre* males by chromosome spreading and distinguished the leptotene, zygotene, pachytene, and diplotene stages with SYCP3 and pH2AX immunofluorescence (**Figure** **3**A). No differences in the distribution of WT and knockout mice in the four pro‐meiotic stages were observed (Figure 3B). In addition, we isolated and evaluated spermatocytes from the testes of 6‐week‐old WT and *Cnot4^fl/fl^;Stra8‐Cre* males by flow cytometry. The percentages of indicated cell types were comparable between the WT and *Cnot4* KO groups (Figure S3G, Supporting Information). The identity and purity of germ cell populations after cell sorting were confirmed by SYCP3 and pH2AX immunofluorescence (Figure S3H,I, Supporting Information). However, *Cnot4*‐null spermatocytes had a significant accumulation of pH2AX at the pachytene stage compared with the WT males (Figure 3A). In *Cnot4*‐null spermatocytes, XY chromosomes were always present in the XY body, suggesting that they paired at the beginning (Figure 3A). In addition, *Cnot4‐*deleted spermatocytes had a high proportion of uncrossed XY chromosomes at the pachytene and diplotene stages (Figure 3A,C). We designed DNA probes to label the X chromosome (green) and Y chromosome (red) and used SYCP3 to label the chromosome axis (Figure 3D). Fluorescence in situ hybridization analysis (FISH) confirmed that the unpaired chromosomes were X and Y chromosomes.

Each pair of homologous chromosomes formed a synapse at the pachytene stage.^[^ ^34^ ^]^ SYCP1, an important component of the synaptic complex, was used as a marker for synapsis. In *Cnot4*‐null pachytene spermatocytes, immunofluorescence and ultra‐high‐resolution microscopy showed that SYCP1 was not present between XY chromosomes, and crossovers were not formed (Figure 3E). The percentage of SYCP1‐negative XY chromosomes was quantified (Figure 3F). After the recombination of homologous chromosomes in the pachytene stage, at least one crossover is formed on the paired chromosomes to ensure accurate cell division. *Cnot4* knockout did not affect the total number of MLH1 foci in pachytene cells (Figure 3G–I). MLH1 localizes at the intersection region and is frequently used as a marker for crossovers.^[^ ^35^ ^]^ However, nearly 60% of the pachytene cells did not form crossovers between XY chromosomes after *Cnot4* knockout (Figure 3J).

Collectively, these results indicate that in *Cnot4*‐null spermatocytes, the XY chromosomes pair at the beginning, but some of them segregate later because crossovers are not formed (Figure 3I).

### 2.5 *Cnot4^fl/fl^;Stra8‐Cre* Males have Defects in DNA Double‐Strand Break Repair

**Figure 4:**
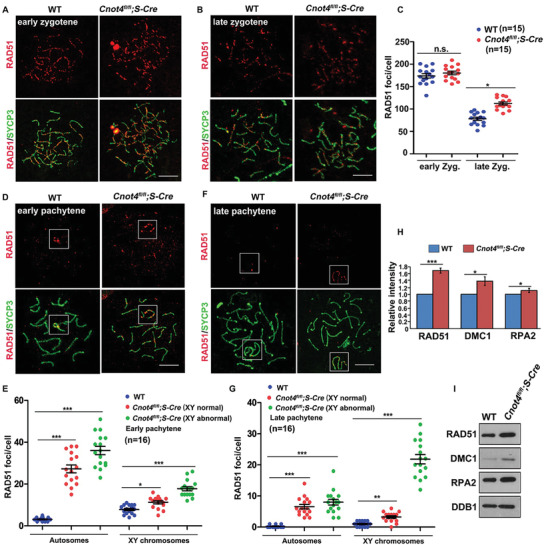
Defective double‐strand break (DSB) repair in spermatocytes of *Cnot4^fl/fl^;Stra8‐Cre* males. A,B) Immunofluorescence staining of RAD51 on chromosomes of WT and *Cnot4*‐null spermatocytes at the zygotene stage. Scale bar = 10 µm. C) Quantification of the RAD51 foci in each cell. D,E) Immunofluorescence staining of RAD51 on the chromosomes of WT and *Cnot4*‐null spermatocytes at the pachytene stage. XY chromosomes are framed. Scale bar = 10 µm. F,G) Quantification of the RAD51 foci on the autosomes and XY chromosomes at the pachytene stage. H) Quantification of the western blot in (I). I) Western blot of RAD51, DMC1, and RPA2 in pachytene spermatocytes isolated from the testes of 6‐week‐old WT and *Cnot4^fl/fl^;Stra8‐Cre* mice. Endogenous DDB1 was blotted as a loading control. *n*: number of cells; error bars, SEM; n.s., non‐significant; **p < 0.01; ***p < 0.001 determined using two‐tailed Student's *t*‐tests.

Immunofluorescence analysis demonstrated that compared with WT spermatocytes, *Cnot4*‐null pachytene spermatocytes had an increased number of pH2AX foci (marker of unrepaired DSBs) on the chromosomes (Figure 3A). We detected other DSB markers in both WT and *Cnot4*‐null spermatocytes. At the early zygotene stage, WT and *Cnot4*‐deleted spermatocytes contained many RAD51 foci (**Figure** **4**A). However, at the late zygotene stage, more RAD51 foci remained in *Cnot4*‐deleted spermatocytes (Figure 4B,C). In *Cnot4*‐deleted spermatocytes, RAD51 foci decreased after the early pachytene stage, and the remaining RAD51 signals were mostly localized on XY chromosomes (Figure 4D,E). In *Cnot4*‐deleted pachytene spermatocytes, more RAD51 foci remained on the autosomes and even more significantly on the sex chromosomes (Figure 4D–G). Western blotting of isolated pachytene/diplotene spermatocytes showed that RAD51 accumulated in the *Cnot4*‐null spermatocytes more than in the WT cells (Figure 4H,I).

The chromosomal foci formed by other proteins localized at the DSBs, including DMC1 (Figure S4A–G, Supporting Information) and RPA2 (Figure S5A–G, Supporting Information) showed a similar pattern as RAD51. Protein expression of DMC1 also increased in *Cnot4*‐null testis lysates (Figure 4H,I). Altogether, these results indicate that DSBs occur in *Cnot4*‐null spermatocytes, but the process is accompanied by an impaired DSB repair process during zygotene‐to‐pachytene transition, particularly on the sex chromosomes.

### 2.6 Non‐Homologous End Joining Signaling Pathway is Impaired in *Cnot4*‐Null Spermatocytes

In the *Cnot4*‐null spermatocytes, there were many unrepaired DSBs at the pachytene–diplotene (PD) stage. There are two DSB repair pathways that are harnessed in the early stage of meiosis in mice. Before early zygotene, DSBs are repaired by homologous recombination (HR). However, from mid‐pachytene to diplotene, DSB repair is mainly mediated by non‐homologous end joining (NHEJ) and involves Ku70 and XRCC4, which are responsible for DSB repair on the autosomes, and 53BP1, which is responsible for DSB repair on the XY chromosomes.^[^ ^36^ ^]^

**Figure 5:**
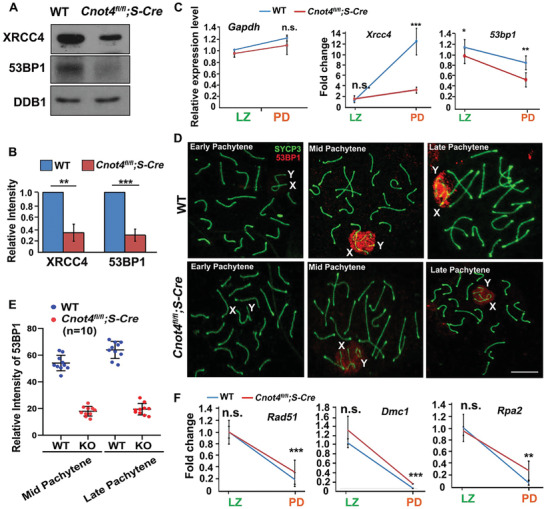
Expression of proteins involved in the non‐homologous end joining (NHEJ) DNA damage repair pathway in spermatocytes. A) Western blotting of XRCC4 and 53BP1 in WT and *Cnot4^fl/fl^;Stra8‐Cre* spermatocytes isolated from the mouse testes by flow cytometry. Endogenous DDB1 was blotted as a loading control. B) Quantification of the western blot results in (A). C) Relative expression of transcripts encoding factors in the NHEJ DNA damage repair pathways measured using RT‐qPCR in isolated spermatocytes. D) Immunofluorescence staining shows expression of 53BP1 in WT and *Cnot4*‐null spermatocytes. E) Quantification of the 53BP1 expression in (D). F) Relative expression of the transcripts encoding the factors in HR DNA damage repair pathways measured using RT‐qPCR in isolated spermatocytes. Error bars, SEM; n.s., non‐significant; **p < 0.01; ***p < 0.001, determined using two‐tailed Student's *t*‐tests.

Western blotting of isolated pachytene spermatocytes showed that expression of XRCC4 and 53BP1 was significantly reduced in the testicular lysates of *Cnot4^fl/fl^;Stra8‐Cre* mice compared with WT mice (**Figure** **5**A,B). This is consistent with the RT‐qPCR results in isolated pachytene spermatocytes that showed decreased mRNA expression of *Xrcc4* and *53bp1* in *Cnot4*‐null spermatocytes (Figure 5C). Immunofluorescence staining indicated that 53BP1 was localized on XY chromosomes in the middle pachytene stage. However, the level of 53BP1 on the XY chromosomes of *Cnot4*‐null spermatocytes was significantly lower than that in the WT spermatocytes at the same stage (Figure 5D,E). In contrast, the genes encoding the proteins of the HR repair pathway, including *Rad51*, *Dmc1*, and *Rpa2*, had higher expression in *Cnot4*‐null spermatocytes than in WT spermatocytes (Figure 5F). This is consistent with the western blot data shown in Figure 4H. These results showed that CNOT4 deletion affects the expression of proteins involved in the DSB repair pathways during spermatogenesis, and CNOT4 deletion promotes the accumulation of unrepaired DSBs, particularly on the X and Y chromosomes in the spermatocytes at the pachytene stage.

### 2.7 CNOT4 is Required to Regulate Transcriptomic Changes from Leptotene–Zygotene to Pachytene–Diplotene Stages in Spermatocytes

To further understand the role of CNOT4 in spermatogenesis at the molecular level, we performed RNA‐sequencing (RNA‐seq) analyses of WT and *Cnot4*‐null spermatocytes at the leptotene–zygotene (LZ) and PD stages isolated by flow cytometry. Gene expression was assessed as fragments per kilobase of transcript per million mapped reads (FPKM). All samples were analyzed in duplicate, and there was high correlation between duplicates (Figure S6A, Supporting Information).

**Figure 6:**
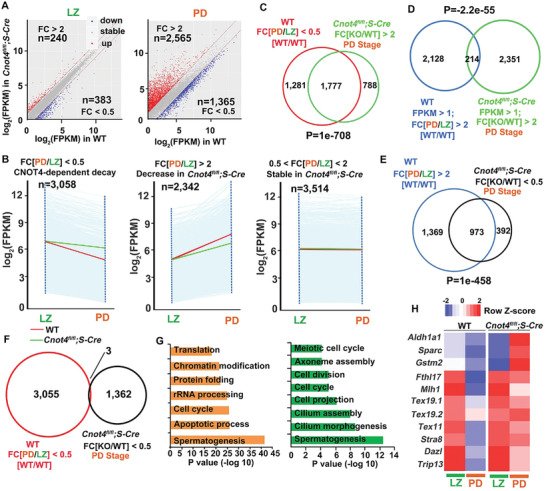
Transcriptomic analyses of the spermatocytes of *Cnot4^fl/fl^;Stra8‐Cre* males. A) Scatter plot of the relative levels of transcripts in the WT and *Cnot4*‐null spermatocytes. Transcripts (*n*) whose levels decreased or increased by more than twofold are highlighted in blue or red, respectively. FC: fold change. B) Dynamic changes in transcripts during spermatogenesis. Transcripts with FPKM > 1 were selected. Each light blue line represents the expression of a single gene, and the middle red and green line represent the median expression of the cluster in the spermatocytes of the WT and *Cnot4^fl/fl^;Stra8‐Cre* mice, respectively. C) Venn diagram of the transcripts whose numbers decreased from leptotene–zygotene (LZ) to pachytene–diplotene (PD) in WT spermatocytes but increased at PD in *Cnot4*‐null spermatocytes. *P* = 1e‐708 determined using two‐tailed Student's *t*‐tests. D) Venn diagram showing transcripts whose numbers increased from LZ to PD in WT spermatocytes and increased at PD in *Cnot4*‐null spermatocytes. *P* = −2.2e‐55 determined using two‐tailed Student's *t*‐tests. E) Venn diagram showing transcripts whose numbers increased from LZ to PD in WT spermatocytes and decreased at PD in *Cnot4*‐null spermatocytes. *P* = 1e‐458 determined using two‐tailed Student's *t*‐tests. F) Venn diagram of transcripts whose numbers decreased from LZ to PD in WT spermatocytes and decreased at PD in *Cnot4*‐null spermatocytes. G) Gene Ontology analysis of transcripts whose levels increased and decreased at PD in *Cnot4^fl/fl^;Stra8‐Cre* mice compared with the WT mice. H) Heatmap of the expression pattern of the transcripts showing increased levels at PD in *Cnot4^fl/fl^;Stra8‐Cre* mice compared with the WT mice. I) Relative mRNA levels of transcripts in LZ and PD stage spermatocytes in WT and *Cnot4^fl/fl^;Stra8‐Cre* mice.

We characterized the transcripts that were differentially expressed during spermatogenesis (FPKM > 1, Figure S6B, Supporting Information). From LZ to PD, 4134 transcripts decreased. The level of these transcripts was significantly higher in *Cnot4*‐null PD spermatocytes than in WT spermatocytes (Figure S5B, Supporting Information). The results of scattergram analyses revealed that 240 and 383 genes were upregulated and downregulated, respectively, by at least twofold in *Cnot4*‐null LZ spermatocytes; in comparison, a larger number of transcripts were deregulated (2565 upregulated and 1365 downregulated) in the PD spermatocytes with *Cnot4* knockout (**Figure** **6**A). This trend became more significant when we increased the fold change cut off to 5 (Figure S6C, Supporting Information). Notably, there were more upregulated transcripts than downregulated transcripts in *Cnot4*‐null spermatocytes at the PD stage (Figure 6A and Figure S6C, Supporting Information).

We divided the transcriptomic changes from LZ to PD in WT mice into three clusters. Cluster I included genes whose transcripts notably declined from LZ to PD (FPKM[PD/LZ] < 0.5, *n* = 3058); included in Cluster II were the genes whose transcripts remarkably increased from LZ to PD (FPKM[PD/LZ] > 2, *n* = 2342); included in Cluster III were the genes whose transcripts remained stable (0.5 < FPKM[PD/LZ] < 2, *n* = 3514) (Figure 6B). Generally, the Cluster I transcripts that decreased from LZ to PD in WT spermatocytes were stabilized in *Cnot4*‐null spermatocytes (Figure 6B). The increase in Cluster II transcripts from LZ to PD was modestly impaired after *Cnot4* deletion. The transcripts in Cluster III were not affected by *Cnot4* knockout (Figure 6B).

The transcripts whose levels increased in *Cnot4*‐null spermatocytes (2565) at the PD stage mainly belonged to Cluster I (Figure 6C), and only a small portion (214) belonged to Cluster II (Figure 6D). On the other hand, among the transcripts whose levels were decreased in *Cnot4*‐null spermatocytes (1365) at the PD stage, 973 belonged to Cluster II and only 3 belonged to Cluster I (Figure 6E,F). Therefore, *Cnot4* deletion mainly prevented the decrease of transcripts that should be removed from LZ to PD and impaired the accumulation of transcripts that should be newly expressed at PD.

Gene ontology analysis revealed that the transcripts that were deregulated at the PD stage after *Cnot4* deletion were related to spermatogenesis, cilium morphogenesis, apoptosis, and cell cycle (Figure 6G). The representative transcripts (reported as essential for spermatogenesis) decreased from LZ to PD in WT spermatocytes and increased from LZ to PD in *Cnot4*‐null spermatocytes (Figure 6H). RNA‐seq also confirmed that the transcripts encoding DNA repair factors, including *Xrcc4*, *Rad51*, and *Dmc1* (Figure S6D, Supporting Information), showed similar expression changes as indicated by the RT‐qPCR results in Figure 5. These results are consistent with those of the testicular phenotypes of *Cnot4^fl/fl^;Stra8‐Cre* mice.

**Figure 7:**
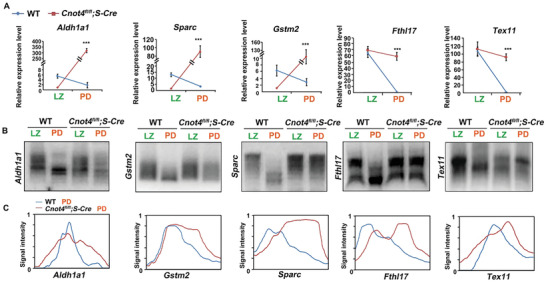
CNOT4 affects mRNA deadenylation and degradation from leptotene–zygotene (LZ) to pachytene–diplotene (PD) stage. A) Relative levels of transcripts in isolated wild‐type (WT) and *Cnot4*‐null spermatocytes measured using RT‐qPCR. B) Changes in poly(A) tail length of transcripts from the LZ to PD stage in isolated WT and *Cnot4‐null* spermatocytes using Poly(A) tail assay. C) Relative signal intensity (*y*‐axis) and length of the PCR products based on mobility (*x*‐axis).

We investigated whether the increase in the number of transcripts in *Cnot4*‐null spermatocytes at the PD stage was due to the long poly(A) tail at the 3′‐UTR of the transcripts. RT‐qPCR results indicated that the mRNA expression of the representative transcripts, as shown in Figure 6H, decreased in WT spermatocytes at LZ to PD and significantly increased in *Cnot4*‐null spermatocytes at PD (**Figure** **7**A). Next, we directly measured the poly(A) tail of the transcripts using a poly(A) tail assay. The poly(A) tails of these transcripts were remarkably shortened from the LZ to PD stages in WT spermatocytes, whereas the shortening was blocked in *Cnot4*‐null spermatocytes (Figure 7B,C). These findings reveal that specific transcripts are removed during the zygotene to pachytene transition following the shortening of the poly(A) tails and that CNOT4 is required for this process.

### 2.8 Short Coding Sequences and 3′‐UTRs are Features of CNOT4‐Regulated Transcripts in the Spermatocytes

We sought to understand the features of transcripts that could be used for identifying degradation or the accumulation of transcripts in spermatocytes at the pachytene stage. Analysis of transcripts revealed that the length of the 5′‐UTR increased in transcripts that were downregulated during the zygotene‐to‐pachytene transition, whereas the length of the 3′‐UTR decreased in transcripts that were upregulated in the same samples (Figure S7A, Supporting Information). The transcripts downregulated in *Cnot4*‐null spermatocytes at the pachytene stage had relatively long 5′‐UTRs, whereas the transcripts that remained unaffected after *Cnot4* knockout at the pachytene stage had long coding sequences and 3′‐UTRs (Figure S7B, Supporting Information). The 3′‐UTR contains many types of consensus regulatory sequences for RNA‐binding proteins (RBPs). The transcripts that were unaffected after *Cnot4* knockout had fewer polyadenylation signals in their 3′‐UTRs, and the transcripts whose numbers decreased in *Cnot4*‐null spermatocytes had fewer AU‐rich elements (Figure S7C, Supporting Information). However, the consensus binding sequence of CNOT4 (GACAGA) was not enriched in the 3′‐UTRs of transcripts that were degraded in a CNOT4‐dependent manner (Figure S7D, Supporting Information).

TUT4/7‐mediated mRNA terminal uridylation is also required for the degradation of a subpopulation of transcripts in pachytene spermatocytes. Transcripts that decreased from LZ to PD in WT spermatocytes overlapped with the transcripts that increased at PD stages in *Cnot4*‐null spermatocytes (Figure S7E, Supporting Information). However, neither group notably overlapped with the TUT4/7‐targeted transcripts in pachytene spermatocytes. These results suggest that CNOT4 regulates mRNA stability in spermatocytes by directly or indirectly interacting with other RBPs instead of recruiting substrates. In addition, CNOT4 and TUT4/7 targeted distinct groups of transcripts for degradation in the meiotic prophase.

### 2.9 CNOT4 is an mRNA Adaptor of CCR4‐NOT Deadenylase during Spermatogenesis

**Figure 8:**
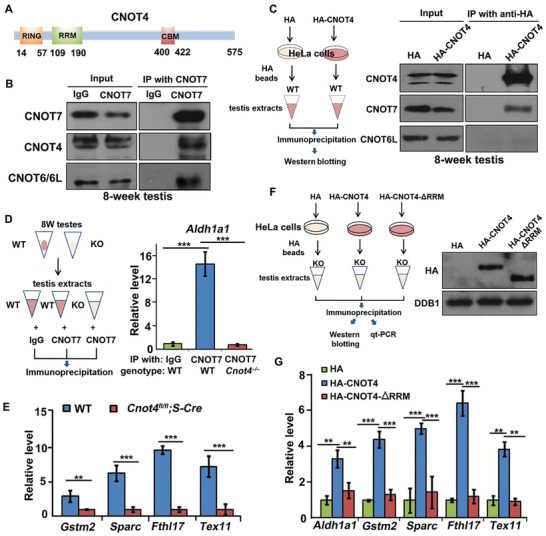
Role of CNOT4 in mediating mRNA deadenylation in the prophase of meiosis. A) Schematic diagram of the domains of mouse CNOT4 protein. RING: RING‐type E3 ubiquitin ligase domain; RRM: RNA recognition motif; CBM: CNOT9‐binding motif. B) Co‐immunoprecipitation and western blotting of endogenous CNOT4 and CNOT7 in the testicular lysate. C) Co‐immunoprecipitation and western blot results of CNOT4 interaction with CNOT7 but not CNOT6/6L in the testicular lysate. D) RNA immunoprecipitation assay for the detection of CNOT7 interaction with mRNAs. E) Interaction of CNOT7 with mRNAs measured using RT‐qPCR. F) RNA immunoprecipitation assay for the detection of CNOT4 binding with mRNAs. Western blotting of HA‐CNOT4 and HA‐CNOT4‐ΔRRM; DDB1 was used as a loading control. G) Relative levels of the mRNAs immunoprecipitated by HA‐CNOT4 and HA‐CNOT4‐ΔRRM, measured using RT‐qPCR.

In vitro biochemical and biophysical studies have indicated that CNOT4 contains an RRM in its N‐terminal region and interacts with the CCR4‐NOT complex through a CNOT9‐binding motif^[^ ^17^, ^37^, ^38^ ^]^ (**Figure** **8**A). In testis lysates, endogenous CNOT4 and CNOT7 were co‐immunoprecipitated by a CNOT7 antibody, suggesting that they were in the same protein complex (Figure 8B). Because there are no commercially available antibodies to pull down endogenous CNOT4, we expressed HA‐CNOT4 in HeLa cells, enriched the protein using HA‐beads, and incubated the beads with testis lysates. Co‐immunoprecipitation indicated that CNOT4 interacts with endogenous CNOT7 but not with CNOT6/6L in the testis (Figure 8C).

As CNOT4 contains an RRM that directly binds mRNA, we speculated that CNOT4 might function as an mRNA adaptor of CNOT7 in mediating transcript clearance during spermatogenesis. ALDH1A1, which is a protein that is specifically expressed in the testes, plays important roles in maintaining low levels of retinoic acid in early testicular development and regulates spermatogenesis after birth. RNA immunoprecipitation indicated that endogenous *Aldh1a1* mRNA coprecipitates with CNOT7 in WT but not in *Cnot4^fl/fl^;Stra8‐Cre* testes (Figure 8D). We tested the interactions of CNOT7 with transcripts that were stabilized in *Cnot4*‐null spermatocytes as shown in Figures 6 and 7. Although these transcripts were higher in *Cnot4*‐null spermatocytes than in WT spermatocytes, they were precipitated at lower levels by endogenous CNOT7 (Figure 8E). This result suggested that CNOT4 mediates the interaction of CNOT7 with its target transcripts.

Next, we tested whether CNOT4 recognizes CNOT7‐targeted mRNAs through its putative RRM. Hemagglutinin (HA)‐tagged CNOT4 and its RRM‐deleted form (ΔRRM) were expressed in HeLa cells and immunoprecipitated with anti‐HA antibody‐conjugated beads. The beads were then incubated with the testis lysates of *Cnot4;Stra8‐Cre* mice, which had minimal levels of endogenous CNOT4 (Figure 8F). RNA immunoprecipitation and RT‐qPCR indicated that these endogenous mRNAs were enriched by HA‐CNOT4 but not by HA‐CNOT4‐ΔRRM (Figure 8G). Therefore, CNOT4 likely associates with mRNA via the RRM.

## 3 Discussion

In eukaryotes, the poly(A) tail is a major determinant of mRNA steady state. Removal of the poly(A) tail by deadenylation is usually the first and rate‐limiting step in mRNA turnover and is important for transcriptome balance.^[^ ^39^ ^]^ According to the canonical working model of poly(A) tail removal, a long poly(A) tail (approximately 250 bp) is initially trimmed by the PAN2‐PAN3 deadenylase complex to ≈150 bp.^[^ ^40^ ^]^ This is followed by a CCR4‐NOT‐mediated deadenylation cycle in which CNOT6 (or CNOT6L) is activated by interactions with cytoplasmic poly(A)‐binding proteins (PABPCs). CNOT6/6L digests PABPC‐bound nucleotides and releases a PAPBC monomer from the tail; the free poly(A) end is then an ideal substrate for CNOT7/8. The degradation by CNOT7/8 removes the free A tail and reactivates CNOT6/6L.^[^ ^5^, ^6^ ^]^ However, the fact that *Cnot6/6l‐*null mice were healthy indicates that this mechanism is dispensable in vivo. In particular, although *Cnot7^−/−^* male mice were infertile,^[^ ^22^ ^]^ the spermatogenesis of *Cnot6/6l^−/−^* male mice was normal, suggesting that the CNOT7 catalytic subunit is sufficient to maintain a functional deadenylation complex when CNOT6/6L is absent. In previous studies, the combination of purified CNOT6 and CNOT7 was required for removing poly(A) tails in an in vitro system,^[^ ^5^, ^6^ ^]^ In contrast, as demonstrated in this study, CNOT7 functions independently of CNOT6/6L in the cellular environment (such as in male germ cells) with the involvement of other regulatory factors such as CNOT4. CNOT6/6L may be an accessory subunit of CCR4‐NOT under certain conditions, such as in maturing oocytes,^[^ ^18^, ^41^ ^]^

CNOT4 is an evolutionarily conserved regulatory subunit of CCR4‐NOT in yeast and humans. However, the importance of CNOT4 in metazoans has not been assessed in vivo, and its role in CCR4‐NOT‐mediated mRNA deadenylation remains unclear. Although studies in yeast and immortalized human cell lines have demonstrated that CNOT4 mediates the degradation of several proteins as a ubiquitin E3 ligase,^[^ ^11^, ^12^, ^13^, ^14^, ^15^, ^16^ ^]^ these CNOT4‐involved biochemical processes are CCT4‐NOT‐independent and unrelated to mRNA stability. In this study, we constructed *Cnot4* knockout and conditional knockout mice. The homozygous *Cnot4* knockout caused embryonic degeneration shortly after implantation. The phenotype of *Cnot4* knockout mice (embryonic lethality) is much more severe than that of *Cnot7^−/−^* mice.^[^ ^18^, ^19^, ^22^ ^]^ This is most likely due to the redundancy of *Cnot7* and *Cnot8* in forming a functional CCR4‐NOT complex during embryonic development. However, *Cnot7* was predominantly expressed in the mouse testis.^[^ ^22^ ^]^ Furthermore, *Cnot7* expression was much higher than that of *Cnot8* in male germ cells. Therefore, the deletion of *Cnot7* was not compensated by the expression of *Cnot8*.

*Cnot4* knockout (*Cnot4^−/−^*) causes embryonic degeneration shortly after implantation, whereas *Cnot4* deletion in male germ cells (*Cnot4^fl/fl^;Stra8‐Cre*) blocks spermatogenesis as early as metaphase I. These phenotypes indicate that CNOT4 is a developmentally indispensable protein in both somatic and germ cells. The spermatogenesis defect in *Cnot7* knockout mice is more extensive than that observed in *Cnot4* conditional knockout mice. As described by Berthet et al.,^[^ ^22^ ^]^ defects in spermatogenesis can be visualized by Sertoli cell vacuolization and tubular disorganization. Because *Cnot7* is deleted in all cell types, including testicular somatic cells, this phenotype could be linked to a defect(s) in germ cells and/or inadequate Sertoli cell function, leading to a complete disappearance of germ cells. In contrast, we deleted *Cnot4* in only male germ cells after meiotic entry, and the function of CCR4‐NOT in Sertoli cells and mitotic germ cells was not affected.

Upon further investigation, we detected an endogenous deadenylation complex containing CNOT4 and CNOT7, but not CNOT6/6L, in spermatogenic cells. CNOT4 and CNOT7 promote the removal of a large number of mRNAs from the zygotene‐to‐pachytene transition in the prophase of meiosis. Experimental evidence suggests that CNOT4 is a bona fide RBP involved in this process and functions as a substrate adaptor of CNOT7 by recruiting the target mRNAs through its RRM. Analyses of the *Cnot4‐*null spermatocytes indicated that CNOT4‐regulated mRNA deadenylation is required for: 1) the appropriate expression of genes involved in the subsequent events of spermatogenesis; 2) timely DSB repair during meiosis; and 3) efficient synapsis and crossover between X and Y chromosomes. The sterility of *Cnot4^fl/fl^;Stra8‐Cre* male mice may be due to the disruption of the spermatocyte transcriptome during the zygotene‐to‐pachytene transition, insufficient expression of genes required for spermatogenesis after the pachytene stage, and build‐up of unrepaired DSBs. The combination of these abnormalities eventually drives spermatocytes to undergo apoptosis before forming spermatozoa. The first role of CNOT4‐mediated mRNA decay is reminiscent of that of BTG4‐ and PABPN1L‐regulated maternal mRNA clearance during oocyte‐to‐embryo transition, which is a prerequisite for zygotic genome activation.^[^ ^20^, ^42^ ^]^ We believe that the second and third roles of CNOT4‐mediated mRNA decay stated above are the significant findings of this study as mRNA stability has not been previously associated with meiosis‐associated DNA repair and sex chromosome pairing, both of which are unique processes of the meiosis prophase. Moreover, these events are sex‐dependent as they are observed only in heterozygous X and Y chromosomes.

*Aldh1a1* is highly expressed in a male‐specific manner in somatic cells in the mouse gonads of developing mouse testes.^[^ ^43^ ^]^ *Aldh1a1* is also expressed in spermatocytes.^[^ ^32^ ^]^ Knockout of *Cnot4* resulted in mRNA accumulation during the LZ‐PD transition process, and *Aldh1a1* was one of the upregulated genes. Therefore, we selected *Aldh1a1* as an example for the RNA immunoprecipitation experiments. As a retinoic acid (RA)‐synthesizing enzyme, ALDH1A1, is expressed in fetal ovaries, providing a source of RA that induces meiosis entry.^[^ ^44^ ^]^ We hypothesized that CNOT4 mediates CNOT7 interaction with *Aldh1a1* transcripts and induces the degradation of *Aldh1a1* in spermatocytes. This might be a mechanism by which *Aldh1a1* expression is downregulated in germ cells.

**Figure 9:**
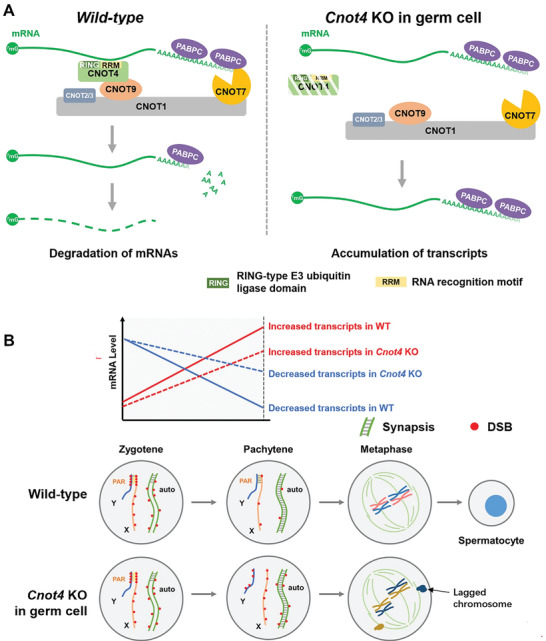
Function of CNOT4 in regulating mRNA stability during the zygotene‐to‐pachytene transition during spermatogenesis. A) Under physiological conditions, a deadenylation complex contains CNOT7 and CNOT4 in male germ cells. CNOT4 as an RNA‐binding protein facilitates CNOT7‐mediated deadenylation and degradation of mRNAs from the zygotene to the pachytene stage of spermatogenesis through its RNA recognition motif (RRM). B) The CCR4‐NOT^CNOT4^‐dependent mRNA removal in the prophase of meiosis is required for 1) the appropriate expression of genes involved in the subsequent events of spermatogenesis; 2) the normal progress of DSB repair during meiosis; and 3) the efficient crossover between X and Y chromosomes. *Cnot4*‐deletion in male germ cells causes the accumulation of transcripts that should have been degraded, leads to defects in DSB repair and XY chromosome pairing, and ultimately results in metaphase arrest and the apoptosis of spermatocytes.

The molecular basis underlying failed DSB repair and abnormal XY chromosome pairing events caused by *Cnot4* knockout require further investigation. Homologous DNA recombination is initiated when 200 to 250 DSBs are generated during meiosis in individual mouse spermatocyte.^[^ ^45^, ^46^ ^]^ As meiosis progresses to the late zygotene stage, most of these DSBs are repaired without the exchange of flanking chromosome arms (non‐crossover), and a subset of DSBs leads to crossovers involving a reciprocal exchange between homologs. Sex chromosomes in males have only a small region of homology, known as the pseudoautosomal region (PAR), which enables pairing; therefore, they are more vulnerable than autosomes to recombination failure.^[^ ^25^ ^]^ DSB formation and repair in PARs are under distinct genetic control and occur later than in autosomes.^[^ ^25^, ^26^ ^]^ *Cnot4* knockout impaired DSB repair, particularly in the sex chromosomes. Moreover, the transcriptomic imbalance caused by *Cnot4* knockout led to the upregulation of proteins in the HR DSB repair pathway and the insufficient expression of proteins of the NHEJ pathway, including XRCC and 53BP1. The NHEJ pathway plays a more important role than HR in repairing later‐stage DSBs and is mainly enriched in PARs.^[^ ^36^ ^]^ It is possible that these persistent DSBs prevent crossover in PARs and lead to inviable gametes (**Figure** **9**). Conversely, a lack of crossover may also cause persistent DSBs.

The pachytene stage lasts seven days in mice and can be divided into early, mid, and late substages because of the drastic chromosome configuration changes. Therefore, any delay in pachytene progression, and hence accumulation of early pachytene spermatocytes, would be reflected in the transcriptome and explain the higher frequency of spermatocytes with unrepaired DSB foci, instead of indicating a direct effect of *Cnot4* deletion. We observed the first wave of spermatogenesis in WT and *Cnot4* conditional knockout mice. In both strains, pachytene spermatocytes were detected as early as postnatal day 17, suggesting that there is no delay in pachytene entry in *Cnot4* conditional knockout mice. In addition, the proportions of spermatocytes at the four stages of meiotic prophase did not change after *Cnot4* knockout, suggesting that pachytene progression is not significantly delayed (Figure 3B).

Although we cannot rule out the possibility that CNOT4 may regulate these processes through mechanisms unrelated to CCR4‐NOT, it is highly likely that the abnormal accumulation of transcripts is a major causal factor, as deletion of *Cnot7* and *Tut4/7* also causes severe defects in meiosis I.^[^ ^22^, ^29^ ^]^ According to the currently accepted models, CNOT7 is the effector of CNOT4‐mediated mRNA poly(A) shortening, and CCR4‐NOT‐trimmed mRNAs are shuttled to TUT4/7 for further modification and degradation.^[^ ^31^, ^41^ ^]^ Nevertheless, the results of our transcriptomic analyses indicate that *Cnot4* deletion causes a more significant accumulation of transcripts compared with the *Tut4/7* knockout.^[^ ^29^ ^]^ Furthermore, CNOT4 and TUT4/7 do not target the same transcripts, and their targeted transcripts have feature differences related to their length and regulatory elements in the 3′‐UTRs. Therefore, CNOT4 and TUT4/7 are required for the clearance of distinct subpopulations of transcripts, and both are essential for spermatogenesis.

The CCR4‐NOT complex promotes mRNA degradation at specific stages during spermatogenesis. In this process, specific mRNA‐binding proteins must be recruited to identify appropriate mRNAs for degradation. In male mice, NANOS2 binds to CNOT1, and with the assistance of another RBP, namely DND1, induces the degradation of specific mRNAs in spermatogonial stem cells.^[^ ^47^, ^48^ ^]^ Male mice with homozygous *Nanos2* knockout lack germ cells in the testes.^[^ ^48^ ^]^ In the final stage of spermatogenesis, the catalytic subunit CNOT7 in the CCR4‐NOT complex may form a piRNA‐induced silencing complex (pi‐RISC) to promote deadenylation and degradation of large amounts of mRNAs during spermiogenesis.^[^ ^49^ ^]^ However, metaphase I arrest of *Cnot4*‐null spermatocytes prevented us from evaluating the potential requirement of CNOT4 in piRNA‐mediated mRNA decay during the final morphogenesis of male germ cells.

The RRM of CNOT4 was reported to recognize the specific sequence GACAGA on mRNA (http://cisbp‐rna.ccbr.utoronto.ca/TFreport.php?searchTF=T39925_0.6). However, this sequence is not enriched in mRNAs that are targeted for degradation by CNOT4 during the zygotene‐to‐pachytene transition. We hypothesize that other sequence or structural features of mRNAs, which are recognized by other RBPs, are involved in prophase‐associated paternal mRNA clearance during meiosis. Further investigation is required to identify whether these additional RBPs interact with CNOT4 or other regulatory subunits of CCR4‐NOT.

## 4 Experimental Section

##### Animals

The *Cnot4^fl/fl^* mouse strain (EPD0682_3_G11) was purchased from the Wellcome Trust Sanger Institute. *Cnot6^−/−^* mice were produced using the CRISPR‐CAS9 system. *Cnot6l^−/−^* mice have been previously reported.^[^ ^18^ ^]^ All mouse strains had a C57BL/6J genetic background. The experimental procedures were approved by the Zhejiang University Institutional Animal Care and Research Committee (Approval# ZJU20170014 to HYF), and mouse care was performed in accordance with the relevant guidelines and regulations of Zhejiang University.

##### Isolation of Spermatocytes

We used 6‐week‐old male C57BL/6 mice to prepare spermatogenic cells at different stages. The fluorescence‐activated cell sorting (FACS) was performed according to a published protocol.^[^ ^50^ ^]^ After the removal of the tunica albuginea, the testes were incubated in 5 mL phosphate‐buffered saline (PBS) with collagenase type I (120 U mL^−1^) at 32 °C with gentle agitation for 10 min. The dispersed seminiferous tubules were further digested with 5 mL of 0.25% trypsin and 0.1 mL DNase I (5 mg mL^−1^) at 32 °C for 8 min. The digestion was terminated by adding 0.5 mL fetal bovine serum (FBS). The resulting suspension was passed through a 70 µm cellular filter. After centrifugation, the cells were resuspended at a concentration of 10^6^ cells mL^−1^ in Dulbecco's modified Eagle's medium (DMEM) with Hoechst 33 342 (5 µg per 10^6^ cells) and 5 µL DNase I, followed by gentle rotation for 30 min at 32 °C. Immediately prior to sorting, the cells were stained with propidium iodide (PI) (1 µg per 10^6^ cells) at 25 ± 2 °C.

Cell populations were collected according to their fluorescent label with Hoechst 33 342/PI staining using FACS (BD Bioscience).

##### Histological and Immunofluorescence Analyses

Testes were fixed in Bouin's buffer or 4% paraformaldehyde (PFA), embedded in paraffin, and sectioned. The sections were deparaffinized, rehydrated, and stained with hematoxylin and eosin (H&E). For immunofluorescence analysis, the sections were boiled in 10 mm sodium citrate buffer (pH 6.0) for 15 min, cooled to 25 °C, washed, blocked with 10% donkey serum for 60 min, and incubated with primary antibodies overnight at 4 °C. Then, the slides were washed and incubated with Alexa Fluor 488‐ or 594‐conjugated secondary antibodies (Jackson ImmunoResearch Laboratories) for 1 h. The slides were mounted with DAPI (Molecular Probes) and imaged under a confocal microscope equipped with a camera (Olympus).

For immunohistochemical analyses, incubation with primary antibodies was performed using biotin‐labeled secondary antibodies. The antibodies were further developed using Vectastain ABC kit and 3,3‐diaminobenzidine peroxidase substrate kit (Vector Laboratories, Burlingame, CA, USA). The slides were then counterstained with hematoxylin and mounted.

##### Western Blotting

Spermatogenic cells at a density of 5 × 10^4^ cells mL^−1^ were lysed in a loading buffer containing 2‐mercaptoethanol and heated at 95 °C for 5 min. Sodium dodecyl sulfate‐polyacrylamide gel electrophoresis (SDS‐PAGE) and western blotting were performed according to standard procedures using a Mini‐PROTEAN Tetra Cell System (Bio‐Rad). Antibodies used are listed in Table S1, Supporting Information.

##### Meiotic Nuclear Spreading and Immunofluorescence Staining

Testis cell spreads were prepared as described previously.^[^ ^51^ ^]^ Seminiferous tubules were treated with hypotonic buffer (30 mm Tris, 5 mm EDTA, 50 mm sucrose, 17 mm trisodium citrate dehydrate, and 0.5 mm dithiothreitol; pH 8.2) for ≈30 min, resuspended, and crushed in 100 mm sucrose buffer (pH 8.2). The suspension was then gently spread onto slides with fixative buffer (1% PFA and 0.15% Triton X‐100; pH 9.2). After 2 h incubation in a moist box, the slides were air‐dried, washed with PBS three times, and stained for immunofluorescence analysis. The slides were blocked with 10% donkey serum and incubated with the primary antibody for 1 h at room temperature. Slides were then washed and incubated with Alexa Fluor 488‐ or 594‐conjugated secondary antibodies and observed using confocal microscopy and ultra‐high‐resolution microscopy (Olympus FVMPE‐RS). Semi‐quantitative analysis of the fluorescence signals was conducted using ImageJ (NIH Imaging program).

##### Metaphase I Chromosome Spreads

MI chromosome spreads were prepared according to a previous report with some modifications.^[^ ^52^ ^]^ Whole testes were suspended in isotonic (2.2%) sodium citrate solution. The suspension was centrifuged, resuspended in 1% sodium citrate solution for 12 min, centrifuged again using the same parameters, and fixed in a 3:1 mixture of absolute ethyl alcohol:glacial acetic acid. After two quick washes in PBS, air‐dried preparations were prepared from the final suspension and stained with Giemsa stain.

##### Fluorescence In Situ Hybridization

Plasmid DNA (chromosome Y: pY353/B; chromosome X: DXWas70) was labeled with SpectumRed dUTP (Vysis, 30–803400) or SpectrumGreen dUTP (Vysis, 30–803200) using a Bioprime DNA labeling system (Invitrogen, 18 094). Spermatocytes separated by FACS were fixed on a glass slide, washed with 2 × SSC (sodium citrate saline buffer) for 10 min, and dehydrated in 80%, 90%, and 100% ethanol for 2 min. The above‐mentioned FISH probe was denatured on a glass slide on a hot plate at 80 °C for 8 min and hybridized in a humid chamber at 37 °C for 48 h. After three washes, the slides were fixed with a fixing medium containing SYCP3. The slides were then washed, incubated with the secondary antibody, and imaged by confocal microscopy.

##### Cell Culture, Plasmid Transfection, and Immunoprecipitation

HeLa cells were cultured in DMEM (Invitrogen) containing 10% FBS (Hyclone) and 1% penicillin–streptomycin solution (Gibco) in a humidified incubator containing 5% CO_2_ at 37 °C. Plasmids were transfected using Lipofectamine 2000 (Invitrogen). After transfection for 48 h, the cells were harvested in a lysis buffer containing 50 mm Tris‐HCl (pH 7.5), 150 mm NaCl, 10% glycerol, and 0.5% NP‐40. The supernatant was immunoprecipitated with gels of different affinities (Sigma‐Aldrich) after centrifugation. Gel beads were washed with lysis buffer after incubation at 4 °C for 4 h. Bead‐bound proteins were eluted using SDS buffer for western blot analysis.

##### Poly(A) Tail Assay

Total RNA was isolated from 10^5^ spermatocytes at different stages using TRIzol. P1 (GCGAGCTCCGCGGCCGCGT_12‐30_) was anchored to Oligo(dT) using T4 DNA ligase. Reverse transcription was performed with oligo(dT)‐anchored P1 using SuperScript IV (Invitrogen). The products were amplified with gene‐specific primers (Table S2, Supporting Information) and the dT anchor primer P1. A0 was amplified using PCR and gene‐specific primers P2 (Table S2, Supporting Information). The PCR conditions were as follows: 30 s at 94 °C, 20 s at 58 °C, and 40 s at 72 °C. The PCR products were electrophoresed on a 2% agarose gel.

##### RNA‐Seq Library Preparation

Spermatocytes at different stages were collected according to genotypes (500 cells per sample) by using FACS. Each sample was directly lysed with 4 µL of lysis buffer (0.2% Triton X‐100, RNase inhibitor, dNTPs, oligo‐dT primers) and immediately used for cDNA synthesis using the Smart‐Seq2 (Illumina) as previously described.^[^ ^53^ ^]^ Sequencing libraries were constructed from 500 pg of amplified cDNA using TruePrep DNA Library Prep Kit V2 for Illumina (Vazyme, TD503) according to the manufacturer's instructions. Barcoded libraries were pooled and sequenced on the Illumina HiSeq X Ten platform to generate 150‐bp paired‐end reads.

##### RNA‐Seq Analysis

RNA‐seq analysis was performed in duplicate using biological replicates for all samples (spermatocytes isolated from testes by flow cytometry). Raw reads were trimmed to 50‐bp reads and mapped to the mouse genome (mm9) using TopHat (v2.1.1) with default parameters. Uniquely mapped reads were subsequently assembled into transcripts using reference annotation (University of California at Santa Cruz [UCSC] gene models) and Cufflinks (v2.2.1). Gene expression was quantified as FPKM, and FPKM values of replicates were averaged. Only the expressed genes (FPKM > 1 in at least one sample) were considered in all analyses. Functional annotation was performed using Metascape (http://metascape.org). Statistical analyses were performed using R (http://www.rproject.org). The Spearman correlation coefficient (rs) was calculated by using the “cor” function, and the complete linkage hierarchical algorithm was used to cluster the genes. Other information on RNA‐seq data used in this study is summarized in Table S4, Supporting Information.

##### RNA Isolation and Quantitative RT‐PCR

Total RNA in spermatocytes was extracted using the RNeasy Mini kit (Qiagen, 74 106) according to the manufacturer's instructions, and was reversely transcribed using the PrimeScript II 1st strand cDNA Synthesis (Takara, 6210A). A random primer (hexadeoxyribonucleotide mixture; pd(N)6;Takara,3801) (50 µM) was used to guide the reverse transcription. RT‐qPCR was performed using Power SYBR Green PCR Master Mix (Applied Biosystems, Life Technologies) and an Applied Biosystems 7500 Real‐Time PCR System. Relative mRNA levels were normalized against endogenous *Gapdh* (internal control). Relative transcription levels were compared with those of the control, and fold change was determined. RT‐qPCR experiments were performed in triplicate. Primer sequences are listed in Table S2, Supporting Information.

##### Statistical Analysis

Statistical data are presented as the mean ± standard error of the mean. Most experiments included at least three independent samples and were repeated at least three times. A two‐tailed unpaired Student's *t*‐test was used to compare the results of the two experimental groups. Using the two‐tailed Student's *t*‐test, *p* < 0.05, *p* < 0.01, and *p* < 0.001 were considered statistically significant and are represented by asterisks (*), (**), and (***), respectively. “n.s.” indicates non‐significant.

## Conflict of Interest

The authors declare no conflict of interest.

## Supporting information

Supporting Information

Supplemental Table 1

Supplemental Table 2

## Data Availability

RNA‐seq data were deposited in the NCBI Gene Expression Omnibus database under the accession code GSE158471. To review GEO accession GSE158471: Go to https://www.ncbi.nlm.nih.gov/geo/query/acc.cgi?acc=GSE158471. Enter token cjypyuyujbwnjeh into the box.
