# Supplementary material for: The CNOT4 Subunit of the CCR4‐NOT Complex is Involved in mRNA Degradation, Efficient DNA Damage Repair, and XY Chromosome Crossover during Male Germ Cell Meiosis
Source: Adv Sci (Weinh). 2021 Mar 16;8(10):2003636. doi: 10.1002/advs.202003636 (PMC8132151; doi:10.1002/advs.202003636)
Supplement: Supplementary file 1 — Supporting Information [file ADVS-8-2003636-s001.pdf]

© 2021 Wiley-VCH GmbH

ADVANCED SCIENCE
Open Access

Supporting Information

for *Adv. Sci.*, DOI: 10.1002/advs.202003636

The CNOT4 Subunit of the CCR4-NOT Complex is Involved in mRNA Degradation, Efficient DNA Damage Repair, and XY Chromosome Crossover during Male Germ Cell Meiosis

Xing-Xing Dai[1], *Yu Jiang*[1], *Jia-Hui Gu*[1], *Zhi-Yan Jiang*[1], *Yun-Wen Wu*[1], *Chao Yu*[2], *Hao Yin*[4], *Jue Zhang*[5], *Qing-Hua Shi*[4], *Li Shen*[1], *Qian-Qian Sha*[3*], *Heng-Yu Fan*[1*]

Supplementary Materials

Supplementary Figures

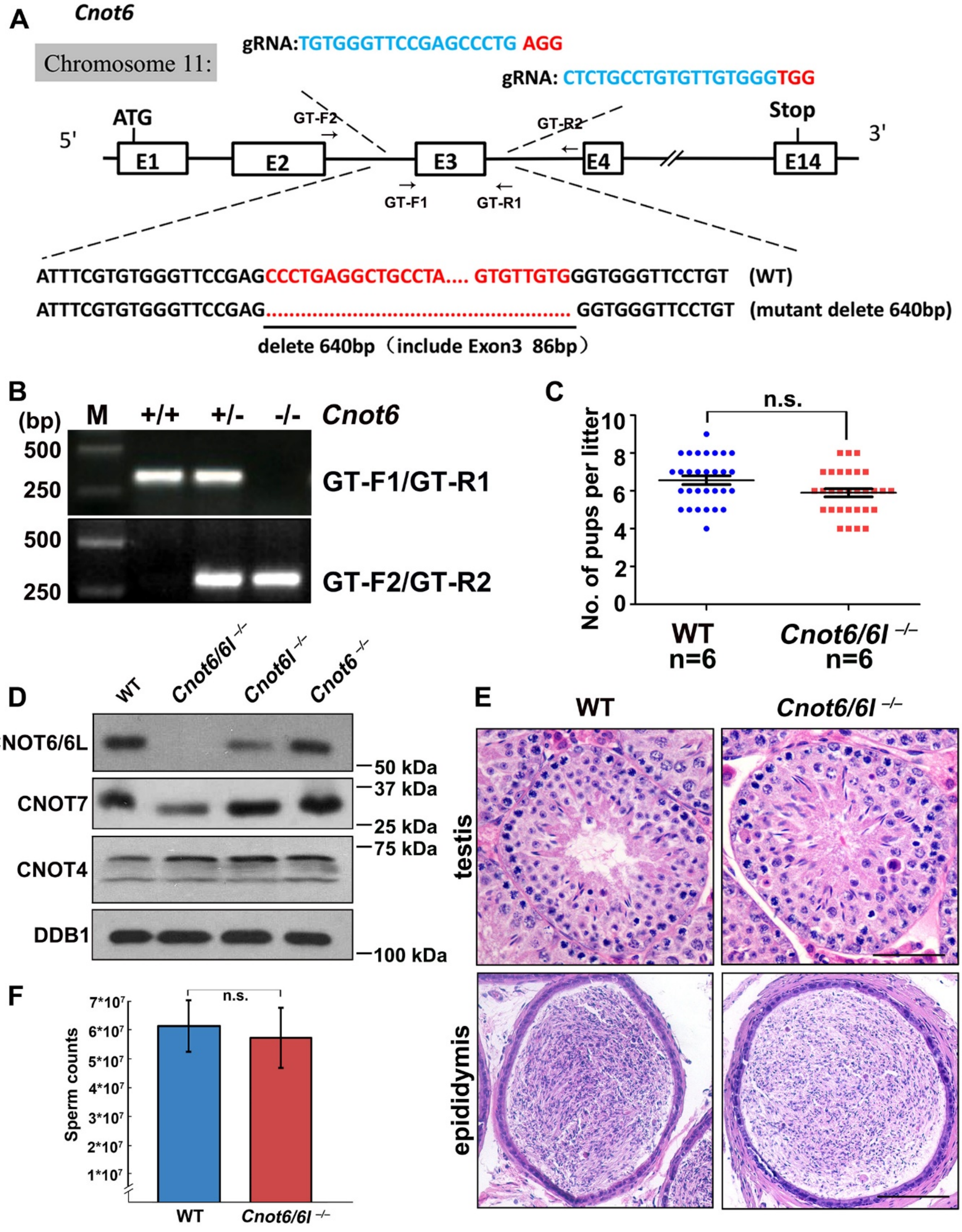

Figure S1. Generation and phenotypic analyses of *Cnot6/6l*$^{-/-}$ mice. A: Gene targeting strategy for CRISPR/Cas9-based mouse *Cnot6* knockout. F, forward; R, reverse; Stop, stop codon; gRNA, guide RNA. **B:** Genotyping of *Cnot6* using mouse tail as DNA template samples. The wild-type (WT) allele ("+") and exon 3 deletion allele ("–") were amplified using the primer pairs GT-F1, GT-R1, and GT-F2, GT-R2. Primer sequences are provided in Supplementary Table S2. **C:** Fertility test results of *Cnot6/6l*$^{-/-}$ and WT males. N = 6 male mice for each genotype; error bars, SEM; n.s., non-significant. **D:** Western blotting of CNOT4, CNOT6/6L, and CNOT7 in the testes of WT, *Cnot6/6l*$^{-/-}$, *Cnot6l*$^{-/-}$, and *Cnot6*$^{-/-}$ male mice. Endogenous DDB1 was used as a loading control. **E:** H&E staining of the testes and epididymis of adult WT and *Cnot6/6l*$^{-/-}$ male mice. Scale bar = 50 µm. **F:** Counts of spermatozoa in the cauda epididymis of 3-month-old WT and *Cnot6/6l*$^{-/-}$ male mice (n = 3 mice for each genotype).

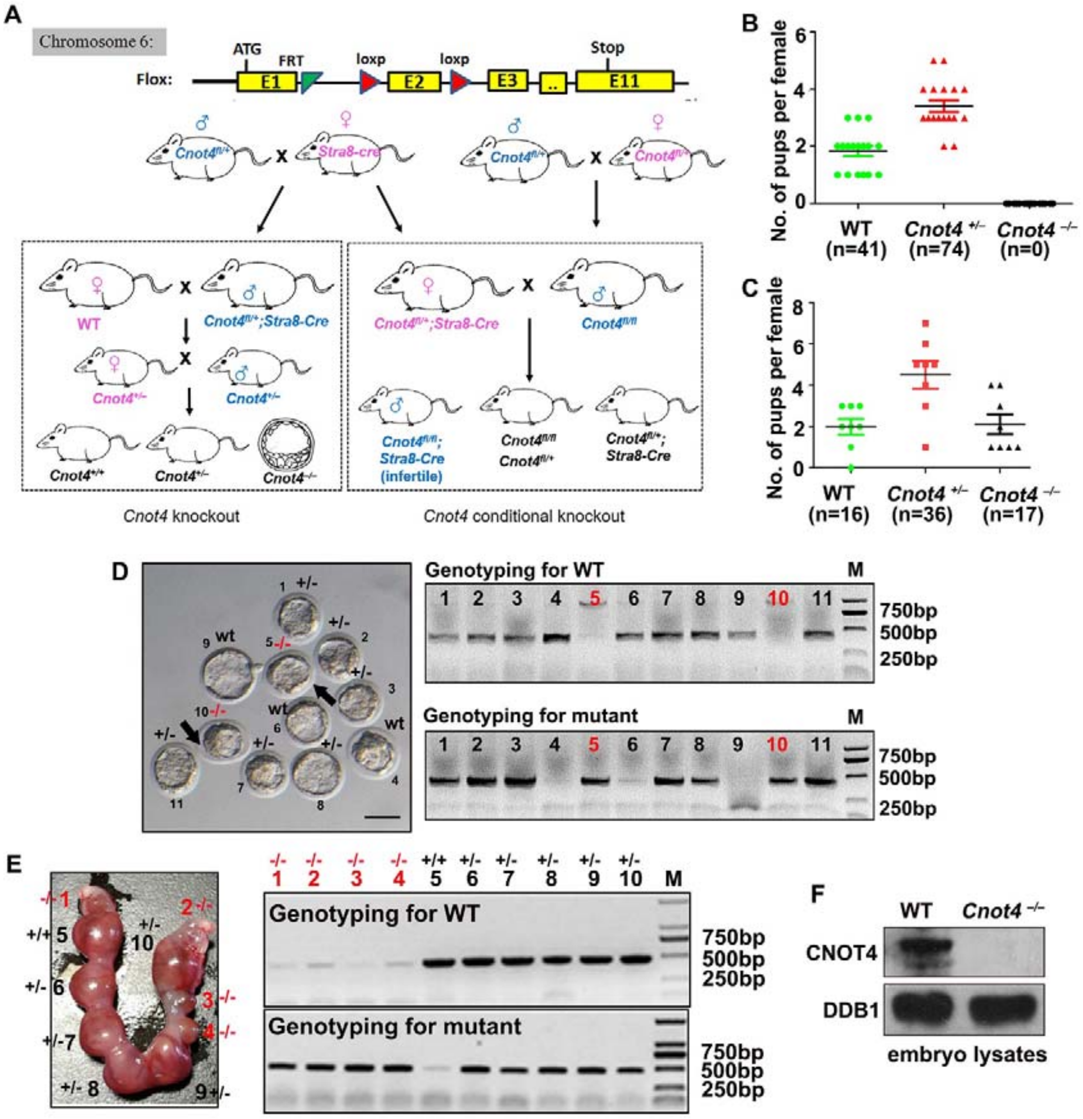

Figure S2. Mouse embryos with homozygous *Cnot4* knockout die during post-implantation development. A: Schematic diagram of the generation of *Cnot4* knockout and conditional knockout mice. **B:** Scatterplot of the genotypes of the offspring produced by mating heterozygous *Cnot4* male and female mice. The numbers of genotyped mice are indicated by *n*. **C:** Genotypes of the offspring. N = 8 female mice. The numbers of genotyped embryos are indicated by *n*. **D:** Morphology and genotypes of 4.5-day blastocysts produced by the mating of *Cnot4*$^{+/-}$ male and female mice. Black arrows indicate *Cnot4*$^{-/-}$ blastocysts. Scale bar = 50 µm. **E:** Morphologies and genotypes of the embryos at the embryonic stage (day 11.5) in uterus. Scale bar = 50 mm. **F:** Western blotting of the CNOT4 in the lysates of 11.5-day WT and *Cnot4*$^{fl/fl}$*;Stra8-Cre* embryos. Endogenous DDB1 was blotted as a loading control.

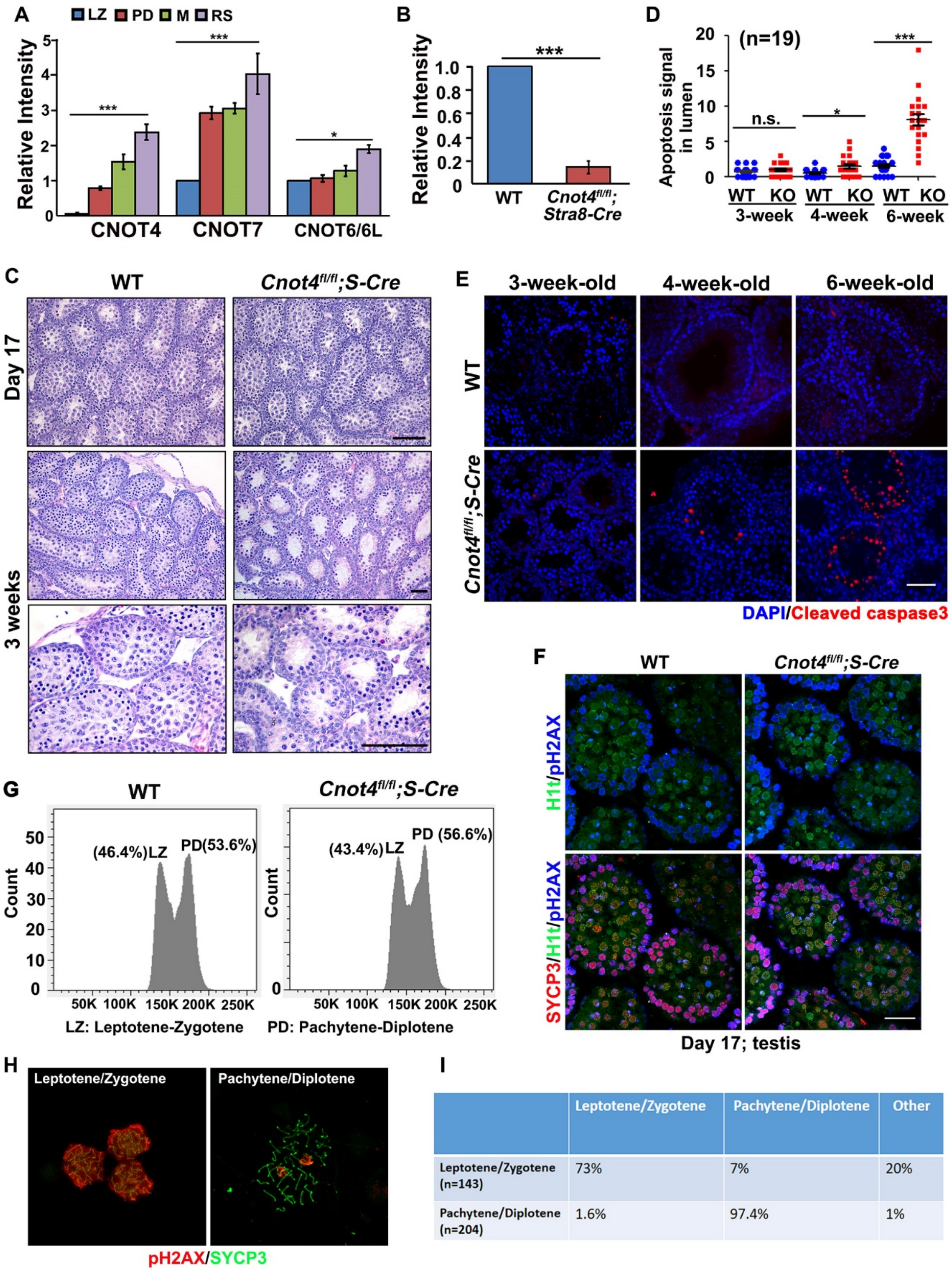

	Leptotene/Zygotene	Pachytene/Diplotene	Other
Leptotene/Zygotene (n=143)	73%	7%	20%
Pachytene/Diplotene (n=204)	1.6%	97.4%	1%

Figure S3. Characterization of meiosis progression in the spermatocytes of WT and *Cnot4$^{fl/fl}$;Stra8-Cre* males. A: Quantification of the western blot in Figure 1C. **B:** Quantification of the western blot in Figure 1H. **C:** Histology of testes from 17-day- and 3-week-old WT and *Cnot4$^{fl/fl}$;Stra8-Cre* mice by H&E staining. Scale bar = 100 μm. **D:** Quantification of apoptotic cells in the lumen of each testicular tubule as shown in (**E**). **E:** Sections of stage IX–X tubules from WT and *Cnot4$^{fl/fl}$;Stra8-Cre* mice stained with cleaved caspase 3. Scale bar = 20 μm. **F:** Sections of the testes from 17-day-old WT and *Cnot4$^{fl/fl}$;Stra8-Cre* mice stained with SYCP1, H1t, and ph2AX. Scale bar = 20 μm. **G:** Cell number counts by flow cytometry showing proportions of spermatocytes at the indicated stages. *Y*-axis indicates the percentage, and the *x*-axis indicates the intensity of DAPI fluorescent signals. **H:** Immunofluorescence of pH2AX and SYCP3 in the leptotene/zygotene and pachytene/diplotene spermatocytes sorted by flow cytometry. **I:** Percentages of cells at the indicated stages in the leptotene/zygotene and pachytene/diplotene spermatocytes sorted by flow cytometry based on the immunofluorescence results presented in (**H**).

Error bars, SEM; n.s., non-significant; **, $P < 0.05$; **, $P < 0.01$; ***, $P < 0.001$ determined using two-tailed Student's *t*-tests.

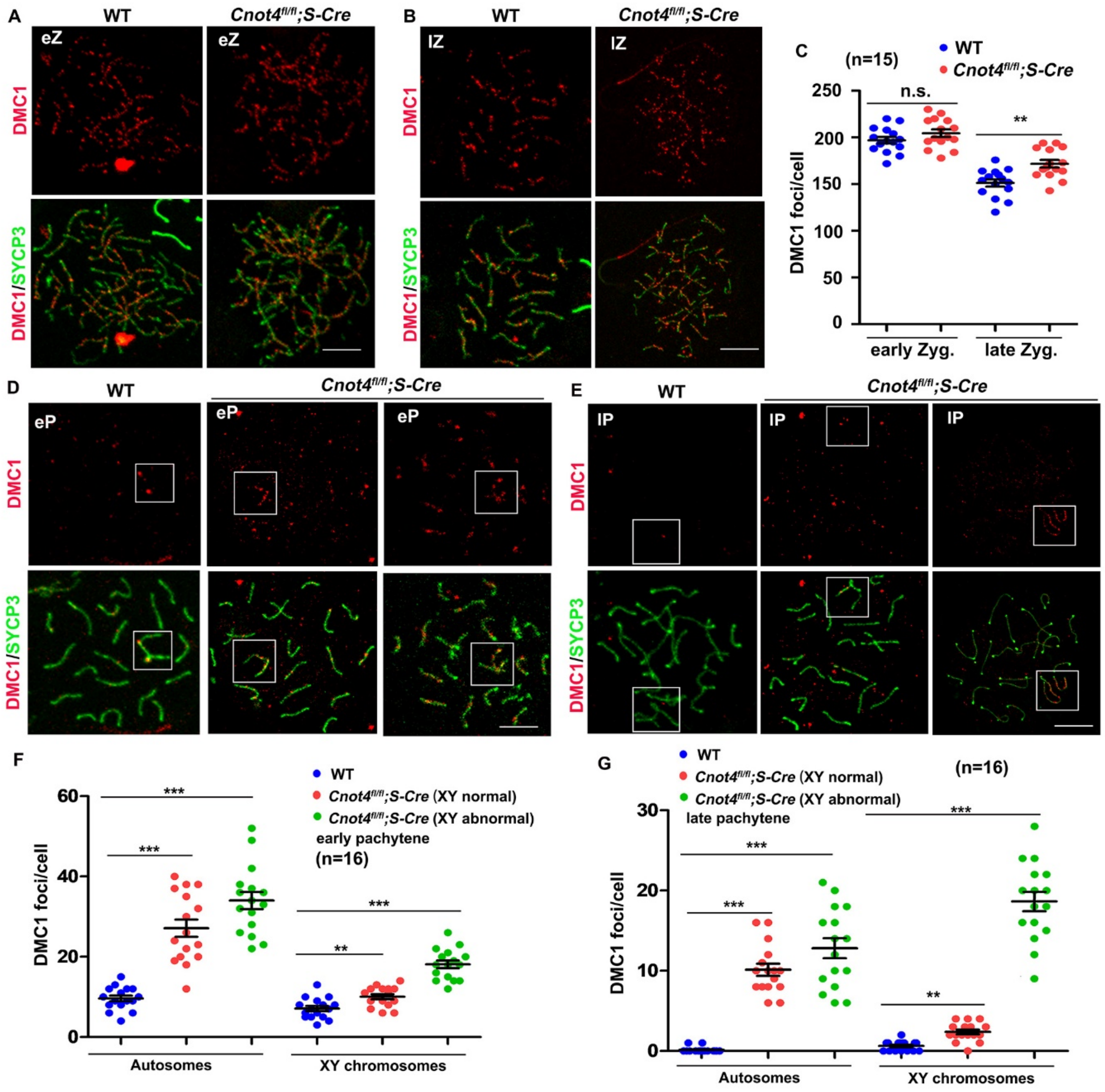

Figure S4. Detection of DMC1 foci in the spermatocytes of *Cnot4*$^{fl/fl}$*;Stra8-Cre* males. **A–B:** Immunofluorescence staining of DMC1 in the chromosomes of WT and *Cnot4*-null spermatocytes at the zygotene stage. Scale bar = 10 µm. **C:** Quantification of the DMC1 foci in each cell. **D–E:** Immunofluorescence staining of DMC1 in the chromosomes of WT and *Cnot4*-null spermatocytes at the pachytene stage. XY chromosomes are framed. Scale bar = 10 µm. **F–G:** Quantification of the DMC1 foci on the autosomes and XY chromosomes at the pachytene stage.

n: number of cells; error bars, SEM; n.s., non-significant; **, $P < 0.01$, ***, $P < 0.001$ determined using two-tailed Student's *t*-tests.

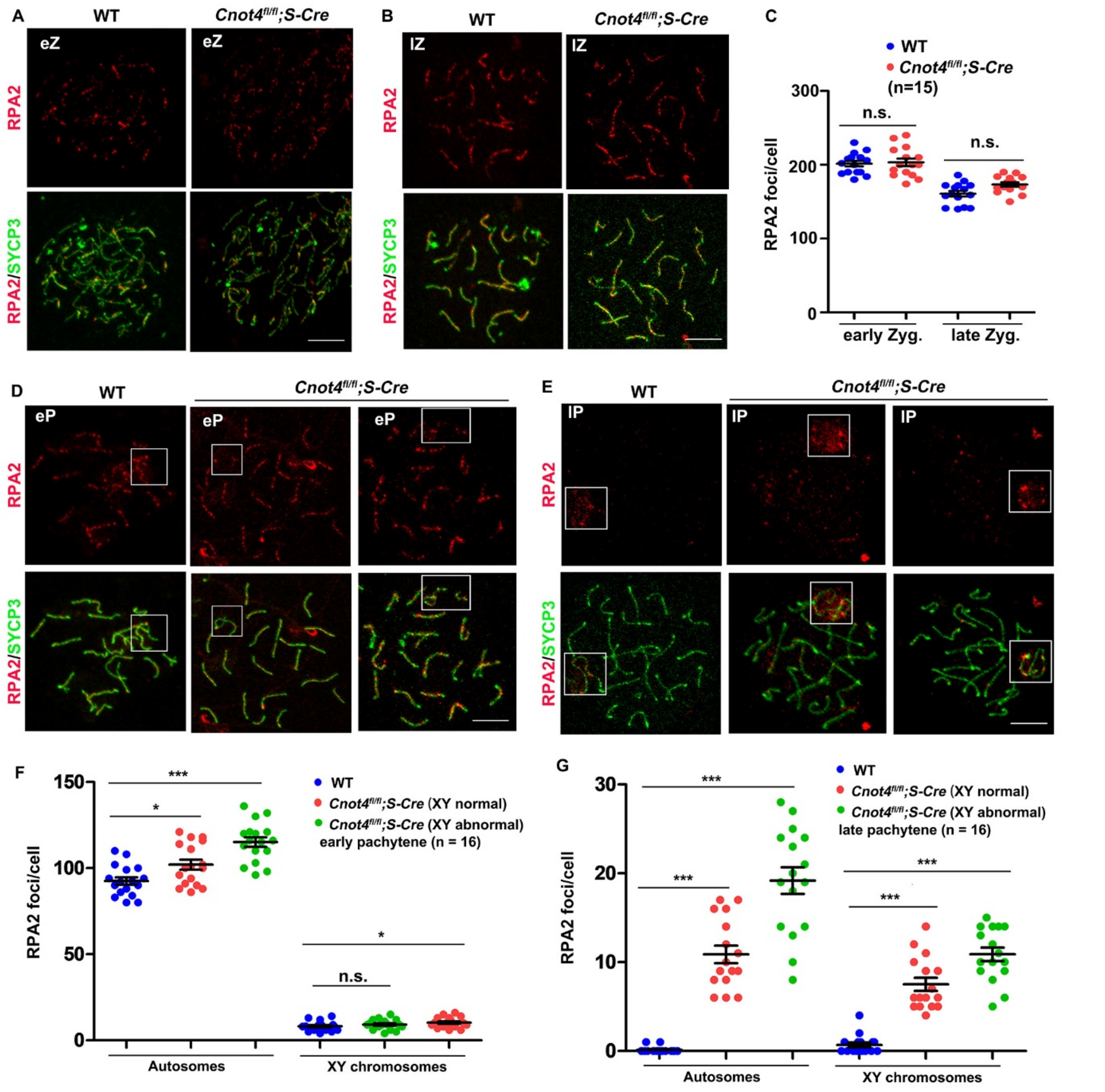

Figure S5. Detection of RPA2 foci in the spermatocytes of *Cnot4$^{fl/fl}$;Stra8-Cre* males. A–B: Immunofluorescence staining of RPA2 in the chromosomes of wild-type (WT) and *Cnot4*-null spermatocytes at the zygotene stage. Scale bar = 10 µm. **C:** Quantification of the RPA2 foci in each cell. **D–E:** Immunofluorescence staining of RPA2 in the chromosomes of WT and *Cnot4*-null spermatocytes at the pachytene stage. XY chromosomes are framed. Scale bar = 10 µm. **F-G:** Numbers of the RPA2 foci in the autosomes and XY chromosomes at the pachytene stage.

n: number of cells; error bars, SEM; n.s., non-significant; **, $P < 0.01$, ***, $P < 0.001$ determined by two-tailed Student's *t*-tests.

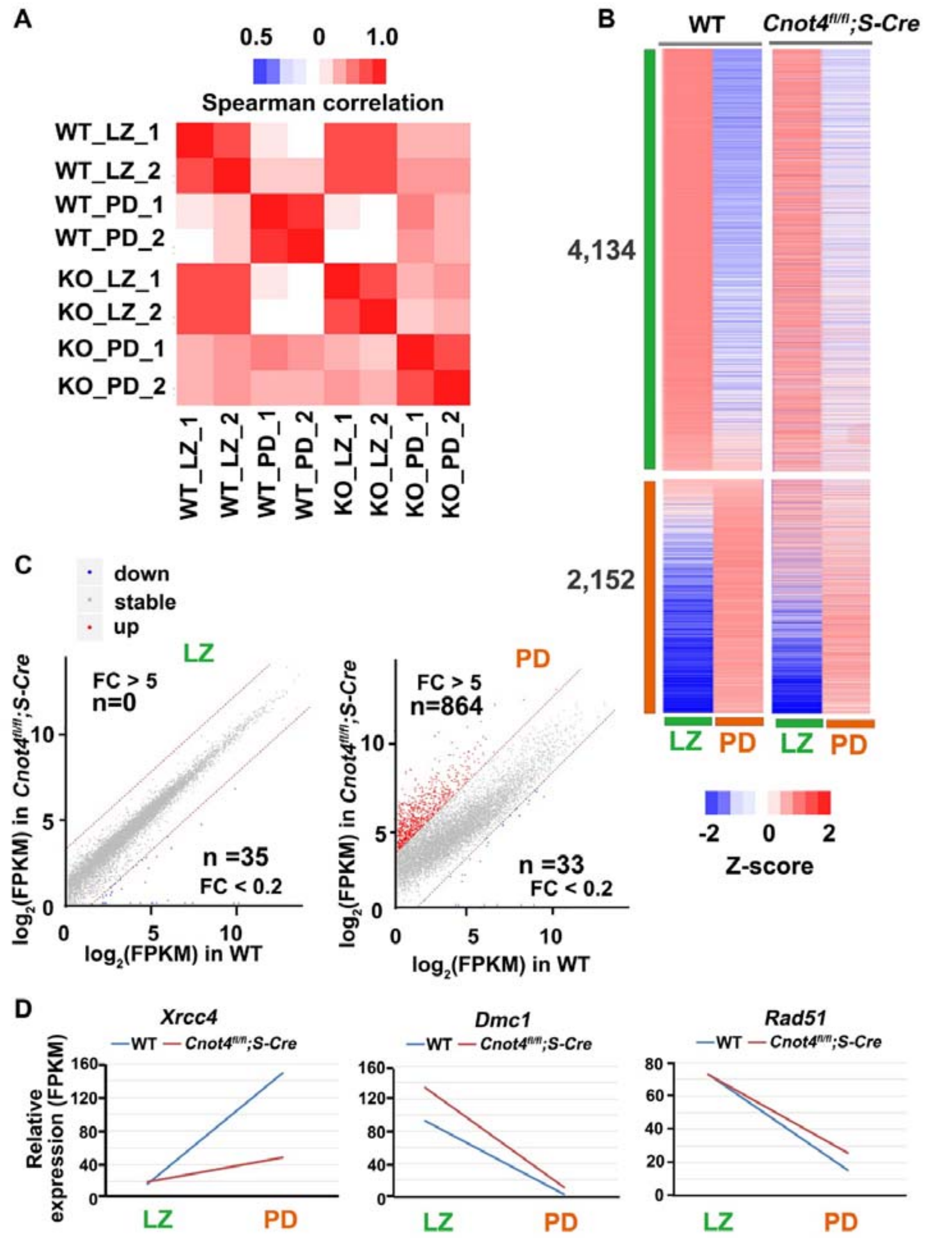

Figure S6. Transcriptomic analysis of the spermatocytes of WT and *Cnot4$^{fl/fl}$;Stra8-Cre* males. A: Heatmap of Spearman's correlation coefficients of the total transcripts between WT and *Cnot4*-null spermatocytes at the leptotene–zygotene (LZ) and pachytene–diplotene (PD) stages. **B:** Heatmap of differentially expressed genes in LZ and PD spermatocytes. The number of identified genes are indicated on the y axis, and the spermatocyte stages are indicated on the x axis. The color key from blue to red indicates relative gene expression from low to high, respectively. **C:** Scatter plot comparing the transcript reads of the WT and *Cnot4*-null spermatocytes. Transcripts (n) that decreased or increased by more than 5-fold are highlighted in blue or red, respectively. FC: fold change. **D:** Relative mRNA expression changes of key mediators involved in DNA double strand repair during meiosis I. The FPKM of each transcript were extracted from the RNA-seq results presented in Figure 6.

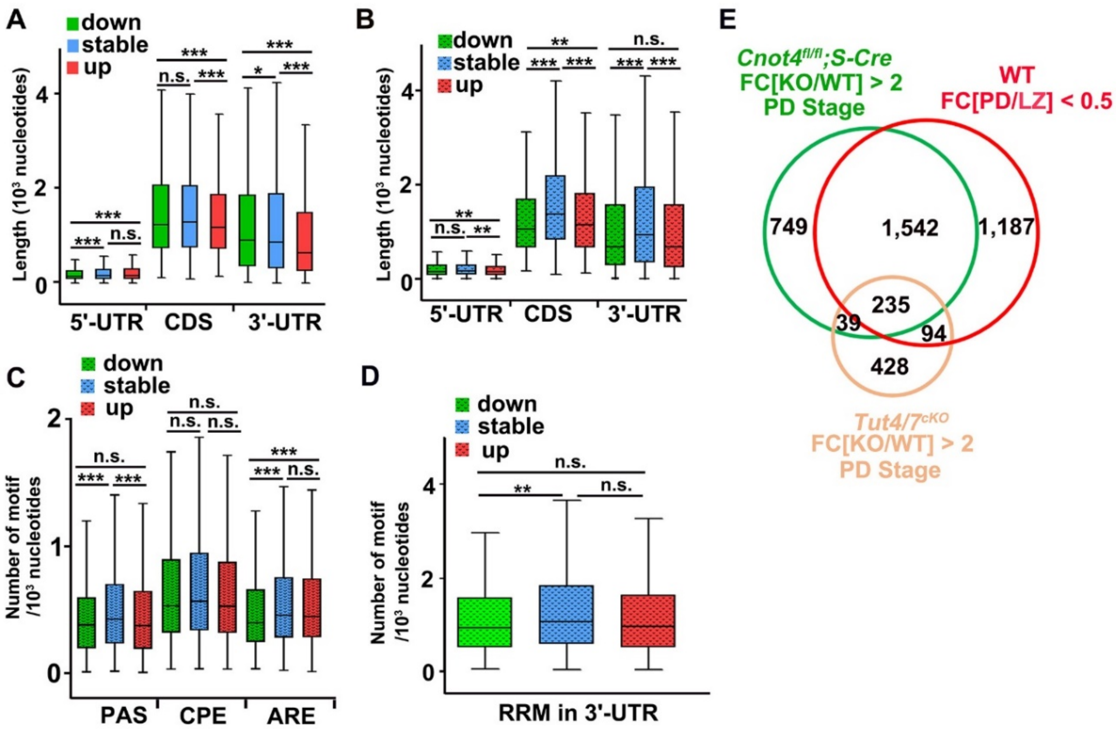

Figure S7. Analyses of CNOT4-targeted transcripts in pachytene spermatocytes. A: Box plot of 5′-UTR, CDS, and 3′-UTR length for downregulated, stable, and upregulated transcripts from the leptotene–zygotene (LZ) to pachytene–diplotene (PD) stage in wild-type (WT) spermatocytes. The center value represents the mean length, and the upper and middle hinges, the first and third quartiles, respectively. **B:** Box plot of 5′-UTR, CDS, and 3′-UTR length for downregulated, stable, and upregulated transcripts at PD stages in *Cnot4*-null spermatocytes. The value at the center represents the mean length, and the upper and middle hinges the first and third quartiles, respectively. **C:** Relative numbers of PASs, CPEs, and AREs in the 3′-UTR of upregulated transcripts in *Cnot4*-null PD stage spermatocytes. The value at the center represents the mean length, and the upper and middle hinges, the first and third quartiles, respectively. **D:** Relative numbers of the sequence recognized by CNOT4 RRM (GACAGA) in the 3′-UTR of upregulated transcripts at the PD stage in *Cnot4*-null spermatocytes. The value at the center represents the mean length, and the upper and middle hinges, the first and third quartiles, respectively. **E:** Venn diagram of transcripts that decreased from LZ to PD in WT spermatocytes but increased at the PD stage in *Cnot4*-null and *Tut4/7*-null spermatocytes.

n.s., not significant; **P* < 0.05; **, *P* < 0.01; ***, *P* < 0.001 determined using two-tailed Student’s *t*-tests.

Supplementary Tables

Table S1. Spearman's correlation coefficients for WT and *Cnot4*-null spermatocytes at the LZ and PD stages.

	Sample		WT				*Cnot4-null*			
Stage	Stage		LZ		PD		LZ		PD	
			1	2	1	2	1	2	1	2
WT	LZ	1	1.00	0.94	0.80	0.80	0.94	0.94	0.83	0.85
		2	0.94	1.00	0.82	0.81	0.94	0.93	0.85	0.86
	PD	1	0.80	0.82	1.00	0.95	0.78	0.76	0.89	0.84
		2	0.80	0.81	0.95	1.00	0.78	0.76	0.87	0.82
Cnot4-null	LZ	1	0.94	0.94	0.78	0.78	1.00	0.94	0.84	0.85
		2	0.94	0.93	0.76	0.76	0.94	1.00	0.81	0.83
	PD	1	0.83	0.85	0.89	0.87	0.84	0.81	1.00	0.93
		2	0.85	0.86	0.84	0.82	0.85	0.83	0.93	1.00

Table S2. FPKMs of transcripts decreased or increased more than 2- or 5-fold in the WT and *Cnot4*-null spermatocytes at the LZ and PD stages (in a separate xlsx file).

Table S3. Antibody information.

Protein name	Manufacture (catalogue number)	Applications (working dilution)	Website Link
**CNOT6/6L**	Abcam (ab86209)	WB (1:500)	https://www.abcam.com/cnot6-antibody-ab86209.html
**CNOT7**	Abcam(ab195587)	WB (1:1000)	http://www.abcam.com/cnot7-antibody-epr18722-ab195587.html
**CNOT4**	Proteintech (12564)	WB (1:500) IHC (1:50)	https://www.ptglab.co.jp/products/CNOT4-Antibody-12564-1-AP.htm
**DDB1**	Epitomics (3821-1)	WB (1:1000)	http://www.epitomics.com/products/search/DDB1
**pH2AX**	Cell Signaling (9718S)	IF (1:400)	https://www.labome.com/product/Cell-Signaling-Technology/9718S.html

**SYCP3**	generated	IF (1:400)	NA
**SYCP1**	Abcam (ab15087)	IF (1:200)	https://www.abcam.com/scp1-antibody-ab15090.html
**MLH1**	Proteintech (11697-1-AP)	IF (1:100)	https://www.ptglab.com/Products/MLH1-Antibody-11697-1-AP.htm
**RAD51**	Abcam (ab176458)	IF (1:100)	https://www.abcam.com/rad51-antibody-chip-grade-ab176458.html
**RPA2**	Huang lab	IF (1:400)	NA
**Cleaved Caspase 3**	Cell Signallin (9664S)	IHC (1:200)<br>IF (1:200)	https://www.cst-c.com.cn/products/primary-antibodies/cleaved-caspase-3-asp175-5a1e-rabbit-mab/9664
**HA**	CST (3724)	WB (1:1000)	https://www.cellsignal.com/products/primary-antibodies/ha-tag-c29f4-rabbit-mab/3724
**FLAG**	Sigma (F3165)	WB (1:3000)	http://www.sigmaaldrich.com/catalog/product/sigma/f3165?lang=zh&region=CN
**XRCC4**	Abcam (ab97351)	WB (1:500)	https://www.abcam.cn/xrcc4-antibody-ab97351.html
**53BP1**	Abcam (ab36823)	WB (1:5000)<br>IHC (1:500)	https://www.abcam.cn/53bp1-antibody-ab36823.html?

Table S4. Quality control of RNA-seq results (WT and *Cnot4*-null spermatocytes at the LZ and PD stages).

Sample	Total reads	Mapping efficiency
WT-LZ-Rep1	12,277,051	80.3%
WT-LZ-Rep2	12,823,790	85.3%
Cnot4-null-LZ-Rep1	13,608,544	84.1%
Cnot4-null-LZ-Rep2	12,008,735	82.0%
WT-PD-Rep1	11,900,426	86.9%
WT-PD-Rep2	13,284,706	87.3%
Cnot4-null-PD-Rep1	11,480,876	82.7%
Cnot4-null-PD-Rep1	14,306,682	82.2%

Table S5. FPKMs of RNA-seq results (in a separate xlsx file).

Table S6. Primer sequences.

**Primer name**	**Genes targeted**	**Sequences (5’-3’)**
GT-F1	*Cnot6*	5’-GAACTCACTCTGTAGACCAG-3’
GT-R1		5’-AGGAAGTCAGAAGACACGTT-3’
GT-F2	*Cnot6*	5’-CAAGAAATAAACCTAAAGGAAAGACTACC-3’
GT-R2		5’-ATGACACGATGAGAAAGCATAGC-3’
Stra8-F	*Stra8*	5’-GTGCAAGCTGAACAACAGGA-3’
Stra8-R		5’-AGGGACACAGCATTGGAGTC-3’
Cnot4 flox-F	*Cnot4*	5’- CTTGCACCTGTGGCTACCAGATATG -3’
Cnot4 flox-R		5’-CTTCAAGACTGTCTATCTCCTTAGTCAGAGAG -3’
P1	N.A.	5’-GCGAGCTCCGCGGCCGCGT12-3’
Gstm2-pat	*Gstm2*	5’-CTCTGGGTCCTTGGGCTCTC-3’
Sparc-pat	*Sparc*	5’-TGGCTGTCATAAAGTTTCTAGCA-3’
Fthl17-pat	*Fthl17*	5’-GCTACCTGACCAACCTGCGC-3’
Tex11-pat	*Tex11*	5’-CCACTGGGCCCTCGCTCCAG-3’
Aldh1a1-pat	*Aldh1a1*	5’-GTCATGACCAGGTGCTTTCCAT-3’
Fthl17-qtpcr-F	*Fthl17*	5’-TCTCGAATGCAGCAGAACTATG-3’
Fthl17-qtpcr-R		5’-GGTCAAAGTAGACTGCCATCG-3’
Tex11-qtpcr-F	*Tex11*	5’-ATGAAGCTCCCTCAACTTTGG-3’
Tex11-qtpcr-R		5’-CCCTCTTTTGCACTTTGTGGA-3’
Aldh1a1-qtpcr-F	*Aldh1a1*	5’-ATACTTGTCGGATTTAGGAGGCT-3’
Aldh1a1-qtpcr-R		5’-GGGCCTATCTTCCAAATGAACA-3’
Gstm2-qtpcr-F	*Gstm2*	5’-ACACCCGCATACAGTTGGC-3’
Gstm2-qtpcr-R		5’-TGCTTGCCCAGAAACTCAGAG-3’
Sparc-qtpcr-F	*Sparc*	5’-GTGGAAATGGGAGAATTTGAGGA-3’
Sparc-qtpcr-R		5’-CTCACACACCTTGCCATGTTT-3’
